# Environmental drivers of *Catostylus tagi* polyp survival and reproduction: unlocking the role of temperature and salinity, supported with citizen science data

**DOI:** 10.7717/peerj.20862

**Published:** 2026-03-17

**Authors:** Pedro F. Silva, João Lopes, Antonina dos Santos, Marcella Saar, Ana Pereira, Paula Enes, Ana S. Ferreira, Hugo Batista

**Affiliations:** 1 Department of Biology, Faculty of Sciences, University of Porto, Porto, Portugal; 2 Oceanário de Lisboa, Lisbon, Portugal; 3 CIIMAR - Interdisciplinary Centre of Marine and Environmental Research, Matosinhos, Portugal; 4 IPMA - Portuguese Institute for Sea and Atmosphere, Lisbon, Portugal; 5 MARE - Marine and Environmental Sciences Centre & ARNET - Aquatic Research Network Associated Laboratory - ISPA - Instituto Universitário, Lisbon, Portugal

**Keywords:** Scyphozoan, Podocysts, Environmental variables, Laboratory study, Community science

## Abstract

**Background:**

*Catostylus tagi* is a scyphozoan jellyfish native to Portuguese waters. While its life cycle is known, the environmental conditions that support polyp survival, trigger strobilation, and promote asexual reproduction remain unclear. Field observations from the citizen science GelAvista project indicate that *C. tagi* occurs year-round in the Tagus estuary, suggesting tolerance to broad temperature and salinity ranges. However, polyps and ephyrae have not been observed in the wild, and their natural habitats and environmental preferences remain unknown. This study aims to fill these knowledge gaps by investigating how temperature and salinity affect *C. tagi* polyps under controlled conditions and integrating these results with field data.

**Methods:**

Ninety-six polyps were cultured for 71 days in well plates at four temperature treatments (14, 17, 20, 23 °C) and four salinity levels (10, 17.5, 25, 35). Survival, asexual reproduction, and strobilation were monitored. Seven years of citizen science data were analysed with environmental parameters to assess estuarine distribution.

**Results:**

Polyp survival was high, except at salinity 35, where mortality increased regardless of temperature. Podocyst production was enhanced at higher temperatures (20–23 °C) and intermediate salinities (17.5–25), although podocyst development into new polyps occurred at lower salinities (10–17.5). Strobilation occurred predominantly at intermediate temperatures (17–20 °C) and salinities (17.5–25). Medusae are most frequently found in the lower Tagus estuary and coastal adjacent areas, exhibiting a peak between July and January. Results showed that wind intensity was negatively correlated with medusa stranding abundances on the shores, possibly displacing individuals away from coastal areas during periods of strong winds. Sea surface temperature (SST), measured two to four months prior to medusae occurrence, was positively correlated with *C. tagi* abundance.

**Discussion:**

Optimal polyp performance occurred at 17–20 °C and 17.5–25 salinity. When considered alongside citizen science observations, these findings suggest that polyps may occur in estuarine areas where such conditions prevail, such as upper estuary inlets and marinas. It is further hypothesized that ephyrae could disperse to downstream and adjacent coastal zones *via* wind and tides. The correlation between sightings and past SSTs indicates the ephyrae and young medusae dispersal rate. Furthermore, this study highlights *C. tagi*’s adaptability to varying environmental conditions and contributes to identifying optimal parameters for polyp well-being and reproduction, with potential applications in jellyfish farming.

## Introduction

Several species of Scyphozoa, particularly those inhabiting coastal environments, exhibit tolerance to a wide range of environmental conditions, including variations in temperature, salinity, pH, oxygen concentration, and food availability (Arai, 1997; Lucas, 2001). Global warming has been implicated in the observed increases in jellyfish populations, leading to changes in bloom size, duration and species distribution. Nevertheless, most scyphozoans exhibit a complex and dimorphic life cycle, alternating between a benthic (polyp) stage and a pelagic (medusa) stage. Medusae reproduce sexually, producing ciliated planktonic larvae called planulae, which settle on a hard substrate, develop, and give rise to polyps. Polyps (*Scyphistoma*) reproduce asexually through various strategies, including lateral budding, stolon formation, and podocyst production, among others, which can result in the formation of hundreds of polyps (Schiariti et al., 2024). Under suitable conditions, polyps undergo strobilation, producing juvenile medusae, called ephyrae, which grow into adult medusae (Lucas, Graham & Widmer, 2012). This remarkably complex life cycle presents significant challenges in determining which life stages are most susceptible to environmental variability (Prieto et al., 2010). Population growth can be attributed to the successful production of ephyrae during the benthic phase, which is typically triggered by environmental factors such as changes in temperature, salinity, or food availability. These factors, however, vary among species (Prieto et al., 2010). Research indicates that increased temperature can enhance polyp density and promote asexual reproduction in certain species (Purcell et al., 2012; Widmer, Fox & Brierley, 2016). Adding to the complexity of this scenario, knowledge about the life cycle of most scyphozoans remains limited, with the medusa stage being more extensively studied than the polyp stage (Mills, 2001; Jarms & Morandini, 2019). Therefore, understanding the factors that influence polyp asexual reproduction, strobilation, and the transition of ephyrae to the adult stage is crucial.

**Figure 1:**
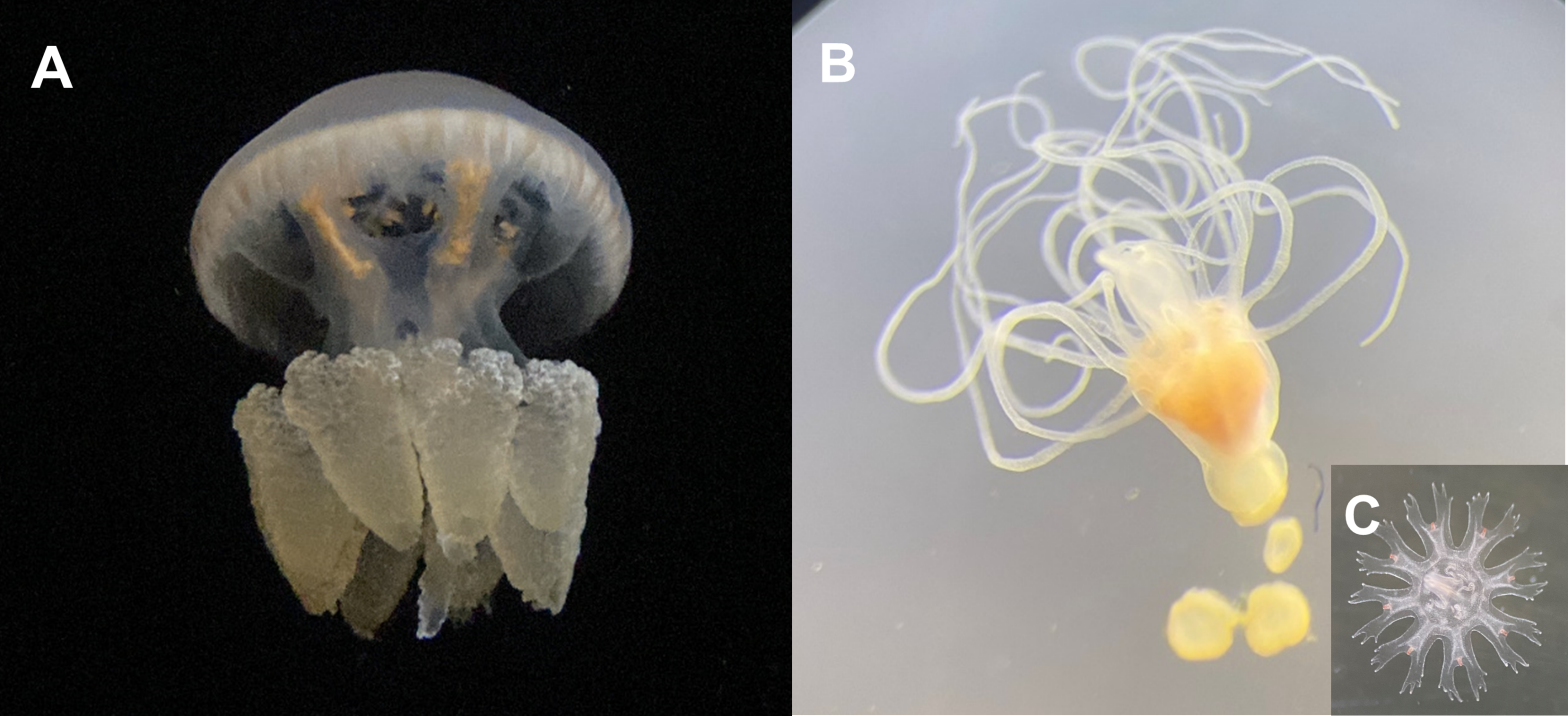
Life cycle stages of *Catostylus tagi*. (A) Adult medusa; (B) Polyp in the pre-strobilation stage with 4 podocysts; (C) Ephyra/Juvenile medusa. Image credits: Pedro F. Silva.

*Catostylus tagi* (Haeckel, 1869) is one of the most common jellyfish species along mainland Portugal and is frequently observed in harbors and marinas, particularly in the Tagus and Sado rivers but also the Algarve coast, the Guadiana river estuary and Ria de Aveiro coastal lagoon, and can be observed throughout the year, especially in the summer months, between August and September (GelAvista, 2024; Gueroun et al., 2021). The medusae of *C. tagi* are typically whitish, brownish, or bluish in color, featuring eight robust oral arms with cluster-shaped excrescences and reddish or brownish fissures along the margin of the bell, which can reach a length of up to 65 cm (Fig. 1A). The polyps have a whitish appearance (Fig. 1B). When fully developed, they possess 16 tentacles used for prey capture and defence (Gueroun et al., 2021). Under specific conditions, such as variations in temperature, the polyps undergo strobilation, releasing ephyrae (Fig. 1C) that develop into adult medusae. Sightings of this species have been collected through the citizen science project GelAvista (gelavista.ipma.pt), which monitors gelatinous organisms in Portugal. Established in 2016, this project engages the community in scientific research, addressing the significant knowledge gaps regarding gelatinous species distribution patterns in Portugal. Data collected by GelAvista indicate that this species is present in the Tagus estuary year-round, with higher abundances during late summer and autumn, suggesting that it exhibits tolerance to a wide range of temperatures and salinities. No sightings of *C. tagi* polyps or ephyrae have been documented in the wild, and their natural habitats remain unknown.

Exhibiting jellyfish in controlled environments provides a valuable platform for public education on marine ecosystems and their conservation (Hayward, 2012). The species *C. tagi* is particularly notable for its local significance in Portugal, offering visitors at the local public aquarium insight into the region’s marine biodiversity. However, gaps in the understanding of this species’ life cycle present challenges in establishing the conditions necessary for the successful *ex situ* reproduction. Furthermore, *C. tagi* has been proposed for biotechnological applications, highlighting the need for reliable methods for species maintenance and reproduction in controlled environments and for the sustainable management of wild populations (Calejo, Morais & Fernandes, 2009).

Gueroun et al. (2021) described the complete life cycle of *C. tagi* following laboratory experiments; however, significant uncertainties remain regarding the polyp stage. Their findings indicate that polyp development is significantly accelerated at higher temperatures (20–25 °C), with the strobilation process occurring 2–3 times faster at 20 °C compared to 15 °C. Additionally, strobilation was found to be influenced by feeding, as unfed polyps did not undergo strobilation. The researchers also observed that temperature did not influence the production of podocysts, the only asexual reproduction strategy observed for this species, leaving unanswered questions regarding the conditions under which podocysts are produced and the timing of their development. While the distribution of adults is well-documented, it does not necessarily indicate that polyps and ephyrae occur in the same locations, nor that these locations provide the optimal conditions for their development and survival. The spatial separation of these life stages complicates the identification of suitable habitats and the assessment of the environmental factors necessary for their survival. In addition to temperature, salinity is likely an important environmental factor influencing the distribution and survival of polyps in coastal regions. However, no studies have specifically investigated the effects of salinity on *C. tagi* polyps, particularly in relation to strobilation, asexual reproduction, or survival. This gap is especially relevant given that salinity and temperature are key environmental factors that influence the annual presence of *C. tagi* medusae. In natural conditions, it is known that several factors, such as seawater temperature (Heim-Ballew & Olsen, 2019), salinity (Widmer, Fox & Brierley, 2016) and wind (Cowen et al., 2007), play a determining role in the reproductive success, development and survival of marine organisms, especially in the larval stage. Nevertheless, most of the available knowledge is limited to tropical and invasive species or species with a major impact on economic activities. Due to the short life span of *C. tagi* and the pronounced fluctuations in its abundance within the Tagus estuary (Gueroun et al., 2021), previous studies have faced significant limitations in characterizing the biological patterns of this species. Furthermore, work on the dynamics of cnidarians on the Portuguese coast is scarce.

This study seeks to examine the responses of *C. tagi* polyps to temperature and salinity conditions characteristic of their natural environment and to leverage citizen science data to enhance our understanding of the species’ ecology within the Tagus region. Specifically, our objectives are to: (1) assess the effects of temperature and salinity on the survival rates of *C. tagi* polyps, as well as on podocyst production and development and on the strobilation process in controlled conditions; (2) optimize culturing methods to identify the conditions that best promote polyp well-being and reproduction under controlled environments; and (3) analyze abundance patterns of *C. tagi* medusae using GelAvista data to identify environmental factors influencing their natural life cycle and connectivity from the upper estuary to coastal areas. Overall, these findings will contribute to a better understanding and management of the species’ life cycle in both controlled and natural environments.

## Materials & Methods

### Ethics statement

*Catostylus tagi* is not a protected or endangered species and is not subject to animal welfare regulations under Portuguese or EU law. All specimens were collected from the Olivais Dock (Lisbon, Portugal), where this species is naturally abundant. The research involved only invertebrate animals and did not require approval from an institutional animal care and use committee. All procedures were conducted in accordance with institutional guidelines for the ethical treatment of marine invertebrates. No specific ethical approval was required for this study.

### Fertilisation

In mid-September 2023, seven adult medusae were collected using a shrimp net at the Olivais Dock, where Oceanário de Lisboa is located. The specimens were placed in buckets and transported to the Jellyfish Laboratory at Oceanário de Lisboa. Since macroscopic identification of the medusae sex is difficult, the gonadal tissue of each medusa was excised, and the sex was determined microscopically. The gonadal tissue was then individually placed in 500 mL containers. Subsequently, male and female tissues were combined and distributed into two buckets, each containing six L of filtered seawater with aeration (temperature: 19 ± 1 °C, salinity: 32.8 ± 0.5), to allow fertilisation. After 48 h, water samples from the buckets were examined under a microscope to verify the presence of planula larvae.

### Polyp maintenance

The planulae were distributed into four aquariums, each with a capacity of eight L, maintained at a temperature of 19 ± 1 °C and salinity of 32.8 ± 0.5, conditions considered favourable for planula settlement (Gueroun et al., 2021). Pre-prepared bioballs, small spherical structures designed to provide a large surface area and covered with biofilm, were introduced into these aquariums, as biofilm presence has been identified as a critical factor for planula attachment (Holst & Jarms, 2007). The bioballs were used to increase the surface area available for planula attachment. Macroscopic observation of settlement began 48 h after the introduction of the planulae. Water parameters, including temperature, salinity, and pH, were monitored daily. Additionally, 50% of the water volume was replaced each morning using a 20 µm sieve to avoid removing planulae from the aquariums. This procedure ensured the maintenance of optimal abiotic conditions and preserved the biological quality of the water. Daily observations of the water were conducted under a stereo microscope to detect the presence of suspended planulae and assess settlement progression. Feeding started late in the morning with six mL of rotifers (at a concentration of 200 rotifers/mL) to nourish the developing polyps. After one week of rotifer feeding, and as a result of polyp growth, the feeding regimen was adjusted to include six mL of freshly hatched *Artemia* nauplii (24 h old, at a concentration of 200 nauplii/mL) administered daily in the late morning, while simultaneously reducing the rotifer supply. The detection of ephyrae marked the onset of the strobilation process, indicating that the polyps had reached a developmental stage capable of undergoing strobilation. To ensure that all polyps remained at the same developmental stage and had not initiated strobilation before the trial, the daily *Artemia* nauplii provision was reduced until the start of the experiment.

### Experimental design and data analysis

**Figure 2:**
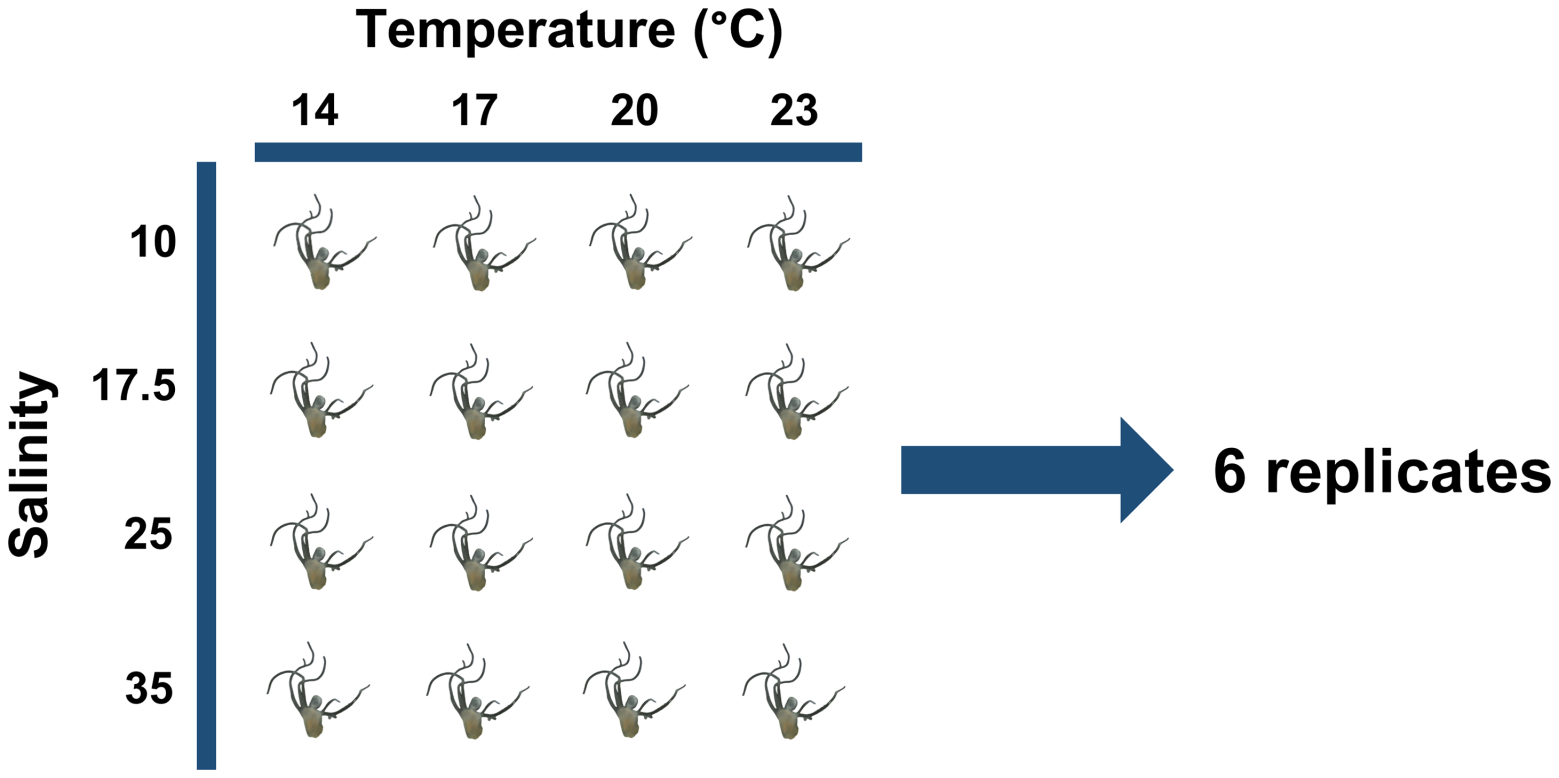
Experimental design showing the temperature (14, 17, 20 and 23 °C) and salinity (10, 17.5, 25 and 35) conditions tested on *Catostylus tagi* polyps.

Two variable experimental factors were studied: Temperature, with 4 levels tested—14 °C, 17 °C, 20 °C and 23 °C and Salinity with also 4 levels tested—10, 17.5, 25 and 35 (Fig. 2). The abiotic conditions chosen are those currently found in the Tagus estuary throughout the year, where *C. tagi* polyps are thought to occur and develop (Rodrigues et al., 2017). To carry out this study, containers were used to maintain the chosen temperatures. Each container was connected to a sump, a reservoir where water is collected and from which it is pumped back into the system, allowing filtered water to recirculate continuously. The set temperatures were achieved using chillers (14 and 17 °C) and water heaters (20 °C and 23 °C). Well plates (each well holding up to 15 mL) were placed in each container. Each well was randomly assigned a salinity, with 6 replicates used for each temperature–salinity combination. The position of the well plates was randomly distributed throughout the containers and remained the same throughout the test.

The wells were filled with water at the chosen salinities and maintained at a temperature of 19 ± 1 °C before the polyps were distributed into the wells (*n* = 1 polyp per well). The polyps were previously acclimated to this temperature in the aquariums to avoid temperature shock. A total of 96 polyps were carefully and randomly removed from the aquariums using a three mL Pasteur pipette. Each polyp was individually observed to ensure that only non-strobilating individuals were selected, as one of the aims of this study is to investigate the abiotic conditions (temperature and salinity) that induce strobilation. The polyps were then distributed into four 100 mL containers, each containing 30 mL of water from their respective aquariums of origin to prevent temperature and salinity shock, thereby minimizing the risk of mortality. To acclimatize the polyps to the four salinities under study, five mL of water at each of the target salinities was gradually added every 5 min to the respective containers, allowing the salinity to approach the desired levels. After acclimatization, the polyps were placed into the experimental system, each in the well corresponding to the assigned salinity. The number of podocysts present on each polyp was recorded prior to placement in the wells in order to estabilish an individual baseline. This was essential to distinguish pre-existing podocysts from those newly produced during the experiment, as one of the objectives of this study was to assess how and under which conditions polyps produce podocysts. After the polyps were placed in the well plates, they were transferred to the respective containers, where the polyps were gradually acclimated to the target temperatures within each well. Since light can influence the variables under investigation, it was ensured that all wells received uniform light exposure under a 12-hour light:12-hour dark cycle. To minimize evaporation and potential contamination, the wells were kept sealed throughout the experiment, except during data collection, feeding, and water changes. Prior to the start of the experiment, the stability of the abiotic conditions (temperature and salinity) inside the wells was verified, confirming that they remained constant when the wells were closed.

During the 71-day experiment, the polyps were fed twice a week in the late morning, *ad libitum*, with 24-hour-old *Artemia* nauplii for 2 h. Following each feeding session, a 50% water change was performed to remove food residues and metabolic byproducts. The 2-hour feeding period ensured that all polyps had the opportunity to feed to satiety, regardless of differences in feeding efficiency or activity at different temperatures. This approach minimized variation in food intake across treatments and avoided potential biases related to enhanced feeding at higher temperatures, which has been previously reported for scyphozoan polyps (Ma & Purcell, 2005). To maintain stable conditions in each well, the water used for both feedings and water changes was pre-prepared to match the salinity and temperature conditions of the respective wells.

**Figure 3:**
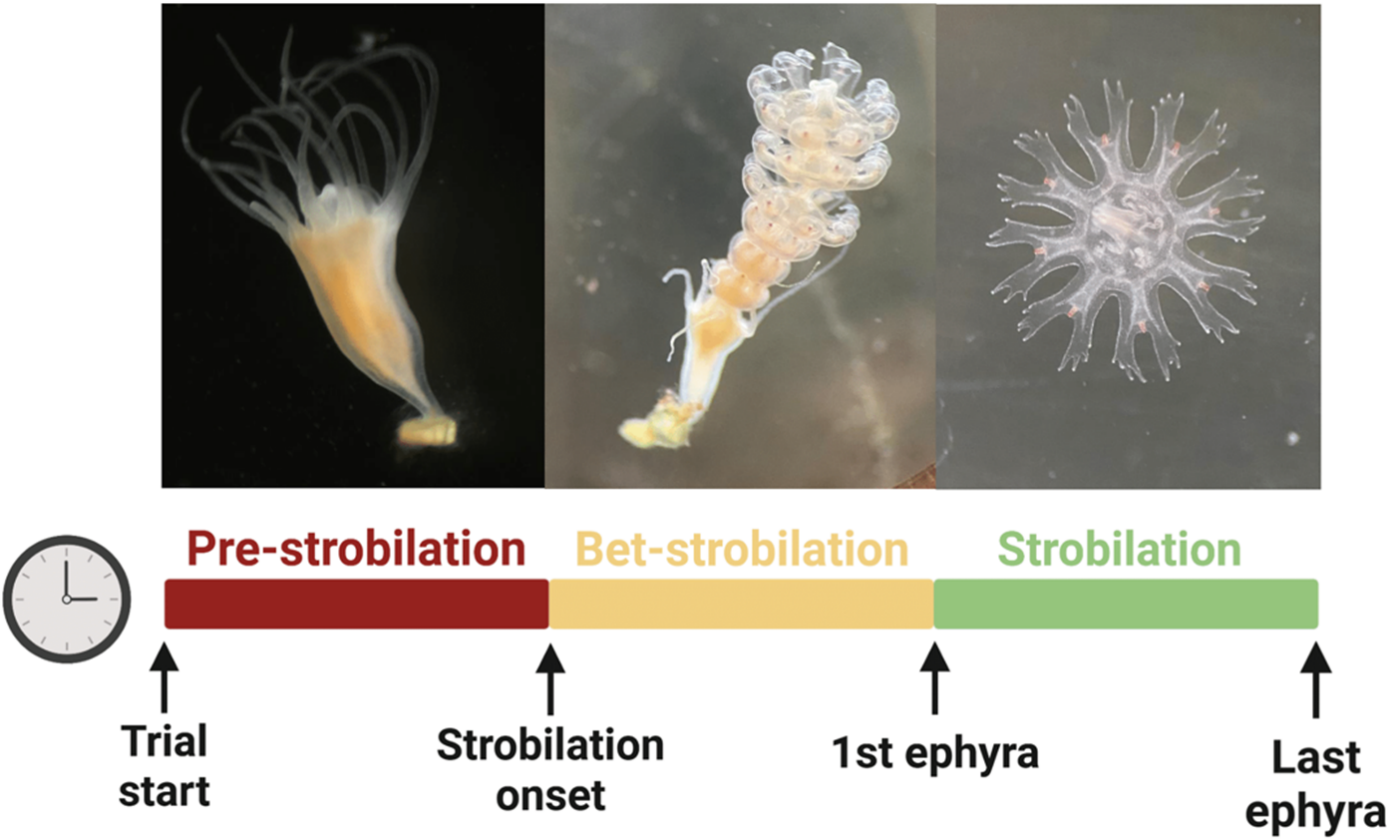
Strobilation process. Image credits: Pedro F. Silva. Figure adapted from Gueroun et al. (2021), presented at the 6th GelAvista Meeting.

The wells containing the polyps were subjected to daily observations under a stereo microscope. The number of polyps that initiated the strobilation process, the number of survivors, the survival duration of the polyps, and the number of podocysts produced and developed were recorded. To gain a deeper understanding of the strobilation process (Fig. 3), three distinct periods were analysed. The ‘pre-strobilation’ period (pre-str) corresponds to the phase during which polyps were not undergoing strobilation. The ‘bet-strobilation’ period (bet-str) represents the interval from the onset of strobilation, defined as the moment when the polyp body begins to undergo morphological changes, characterized by the formation of transverse furrows, until the release of the first ephyra. Finally, the ‘strobilation’ period (str) was defined as the time interval between the release of the first and the last ephyra. The number of ephyrae produced per polyp was quantified and removed during the observations of the wells. Other reproductive strategies, such as budding or longitudinal fission, have been investigated under the different temperature and salinity conditions tested to confirm that podocyst production remains the only recorded reproductive strategy for this species (Gueroun et al., 2021).

The effects of temperature and salinity on polyp survival, survival time, the number of podocysts produced and developed, and the number and duration of strobilation cycles were analyzed using a two-factor analysis of variance (ANOVA). The data were tested for normality and homogeneity of variance prior to analysis using the Shapiro–Wilk and Levene tests, respectively. In the case of interaction between factors, a one-way ANOVA was performed for the variables under study. Tukey’s *post-hoc* tests were used to identify the specific treatments (temperature and salinity) responsible for the observed differences between groups in the ANOVA. Differences were considered statistically significant at *p* < 0.05. All statistical analyses were performed using SPSS software (version 29.0, IBM Corp, Armonk, NY, USA) and the Data S1 to S4 are available in the Supplemental Files.

### Citizen science and environmental data analysis

GelAvista is a citizen science project that requires citizens to engage in scientific research through voluntary observations, with the ability to submit jellyfish sightings *via* a mobile app and a dedicated email. Through these platforms, citizens submit photos and/or videos, for the identification and validation of the species, as well as information about the location, date, time and number of individuals sighted. The information received is validated and the species identified by experts, assigning a level of confidence in the observations (1–3), where 3 has the lowest reliability. In this study, only observations with confidence level 1 (maximum reliability) were considered. This study used all available data provided by GelAvista for the species *C. tagi* collected between January 2016 and December 2022 in the Tagus estuary and adjacent marine environments, which correspond to 1.368 records.

The study area was divided into eight sub-areas following the classification proposed by Rodrigues et al. (2017), which categorises the Tagus estuary based on distinct environmental characteristics and taking into account the population density in the different areas and margins of the Tagus estuary. The northern and southern shores were differentiated, with the northern shore further subdivided into two sections, separated at Cabo Raso (Fig. S1). Due to insufficient records, the uppermost area (area 5) was excluded from the analysis.

Abundance data received by GelAvista are recorded using abundance ranks. For the purposes of statistical analysis, these intervals were conservatively converted to point estimates by incrementing the minimum value of each rank by one (for example, a range of “2 to 5” was represented as three individuals; “6 to 10” as 7; “11 to 50” as 12). For open-ended intervals denoted by “greater than”, a single unit was added to the lower bound (*e.g.*, “>50” was interpreted as 51 individuals; “>100” as 101). Abundance data were calculated as the sum of observations recorded by citizen scientists within each zone and aggregated into monthly intervals. The database for these records is available in Data S5. To mitigate variability in data magnitude, sighting records were transformed using a logarithmic scale, a standard approach when values exhibit significant differences in magnitude (*e.g.*, 1 *vs.* 1.000), as was observed in this study. Log-Abundance sightings information of *C. tagi* was analysed in relation to environmental variables by parametric and non-parametric correlation analyses. Variables considered were: average wind intensity (m/s), average wind direction (°), average weekly precipitation (mm), average air temperature (°C), SST - average sea surface temperature (°C) and average chlorophyll *a* index (mg/m^3^) as a proxy for primary productivity. The wind intensity and direction, precipitation and air temperature were consulted on the IPMA website (https://www.ipma.pt). Sea surface temperature and chlorophyll *a* index were obtained from the NASA website (https://oceancolor.gsfc.nasa.gov). Given that the GelAvista program collects medusae abundance data and temperature is expected to influence the earliest developmental stages, a cross-correlation analysis was performed to assess the influence of temperature, lagged several months, on *C. tagi* medusae abundance.

To conduct the temporal analysis and identify the environmental variables that best explain variations in *C. tagi* abundance, the Generalized Estimating Equation (GEE) statistical method was applied. This approach is particularly suitable for analysing correlated data, time series, and geographically grouped observations, as it accounts for dependencies among observations (Hardin & Hilbe, 2012). The analysis was performed using *C. tagi* abundance per geographical sub-area (as previously described) as the dependent variable. Given the high prevalence of zeros, a negative binomial (with log link) model was employed to improve model fit. Several models incorporating different independent variables as predictors were tested, and the best-fitting model was selected based on the Quasi-likelihood under the Independence Model Criterion (QIC), a statistical criterion used for model selection in regression analysis. The optimal model was identified as the one with the lowest QIC value. All statistical analyses were conducted using IBM SPSS (Statistical Package for the Social Sciences), Version 29.0.0.0 (241).

## Results

The combined results of temperature (14, 17, 20, and 23 °C) and salinity (10, 17.5, 25, and 35) on polyp survival, survival time, the number of podocysts produced and developed, the number and duration of strobilation cycles, and the number of ephyrae produced are presented in Table S1.

### Effects of temperature and salinity on polyp survival

**Figure 4:**
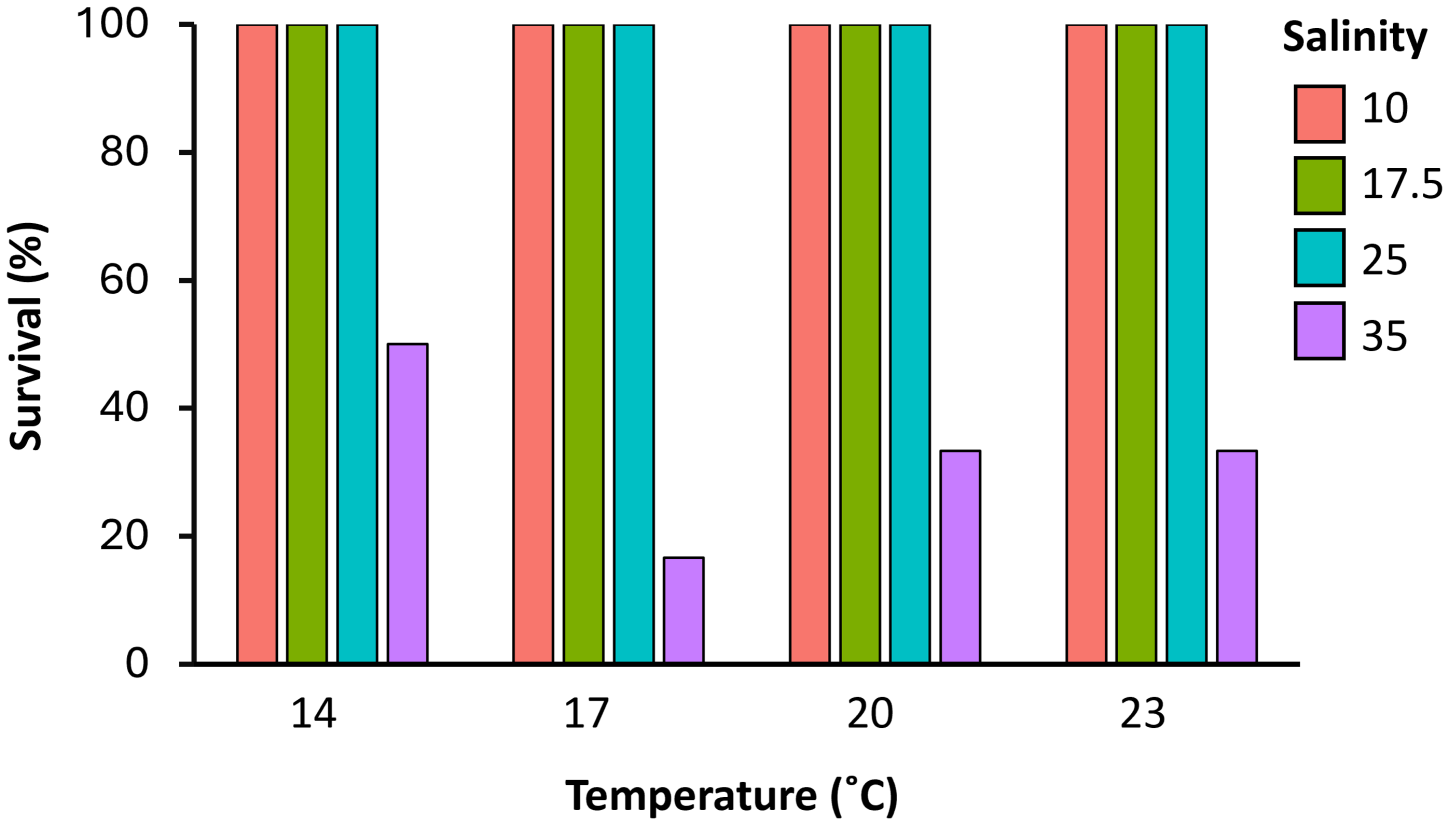
Survival (%) of *Catostylus tagi* polyps maintained under 16 different combinations of four temperatures (14, 17, 20, and 23 °C) and four salinities (10, 17.5, 25, and 35) over a 71-day experiment (*n* = 6 per combination).

Regarding polyp survival and survival time, temperature and the interaction between temperature and salinity had no significant effects (Table 1). However, salinity significantly influenced these factors (*p* < 0.05). Across all tested temperatures (14, 17, 20, and 23 °C), mortality was only observed at a salinity of 35, whereas polyps exposed to salinities of 10, 17.5, and 25 exhibited 100% survival throughout the experimental period (Table S1, Fig. 4). At 14 °C, 50% of polyps exposed to 35 survived throughout the experiment. In contrast, survival rates at 35 declined significantly at higher temperatures, with survival rates of 16.6%, 33.3%, and 33.3% observed at 17, 20, and 23 °C, respectively. Polyps exposed to a salinity of 35, regardless of temperature, initially exhibited reduced activity, as evidenced by a noticeable decrease in movement and overall activity compared to polyps maintained in lower salinity conditions. A further indication of stress was a reduction in feeding behavior, observed either through a lack of interaction with the food provided or by the retraction of the tentacles, which are typically used for food capture. These behavioral changes ultimately led to the death of the polyps, which disintegrated into small fragments scattered throughout the wells.

**Table 1:** Effects of combinations of temperature and salinity on the survival, asexual reproduction, and strobilation of *Catostylus tagi* polyps. (-) Not significant; *P < 0.05; ** *P* < 0.01; *** *P* < 0.001. When significant differences are detected for temperature and/or salinity, different lowercase letters are used to denote which specific categories differ significantly from each other (*P* < 0.05). For example, in the case of polyp survival, only the salinity of 35 (denoted by “a”) differed significantly from the other salinity levels (denoted by “b”).

Variation source	Temperature	Salinity	Interaction	Temperature				Salinity			
				14	17	20	23	10	17.5	25	35
**Polyp survival**	–	***	–	–	–	–	–	b	b	b	a
**Survival time**	–	***	–	–	–	–	–	b	b	b	a
**Podocyst production**	***	***	***	a	a	b	c	ab	bc	c	a
**Podocyst development**	–	**	–	–	–	–	–	b	ab	a	a
**Strobilation (N° Cycles)**	***	***	**	a	b	b	b	a	b	b	a
**Strobilation phases**:											
**Pre-strobilation (pre-strob)**	***	**	***	b	ab	a	a	b	a	ab	ab
**Bet-strobilation (bet-strob)**	***	*	*	c	b	ab	a	a	b	ab	ab
**Strobilation (strob)**	***	–	–	b	b	a	a	–	–	–	–
**Ephyra production**	–	–	–	–	–	–	–	–	–	–	–

### Effects of temperature and salinity on polyp asexual reproduction

**Figure 5:**
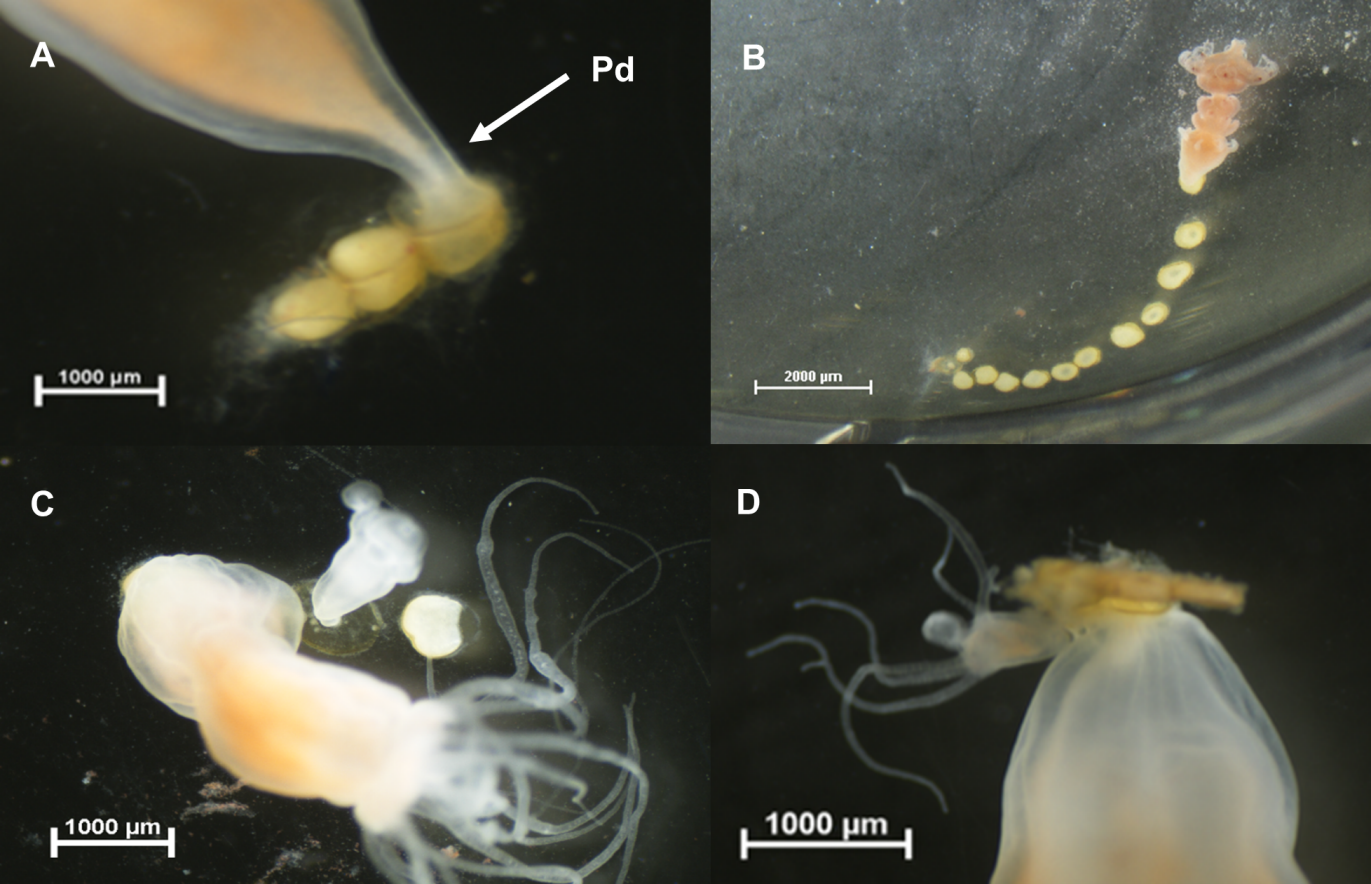
Podocyst formation and development in *Catostylus tagi*. (A–B) Podocysts produced by *C. tagi* Pd –pedal disc. (C–D) Polyp development from podocysts: (C) Newly formed polyp with two tentacles; (D) More developed polyp with five tentacles. Image credits: Pedro F. Silva.

With respect to the asexual reproduction of the polyps, podocyst production was identified as the sole reproductive strategy observed (Fig. 5), with podocysts forming on the pedal discs of the polyps (Fig. 5A). Polyps developed a new pedal disc at the basal part of their bodies, while the old pedal disc was reabsorbed, resulting in the release of the podocyst and the loss of its anchoring function. In some cases, this process led to the formation of podocyst cascades at the bottom of the wells (Fig. 5B).

**Figure 6:**
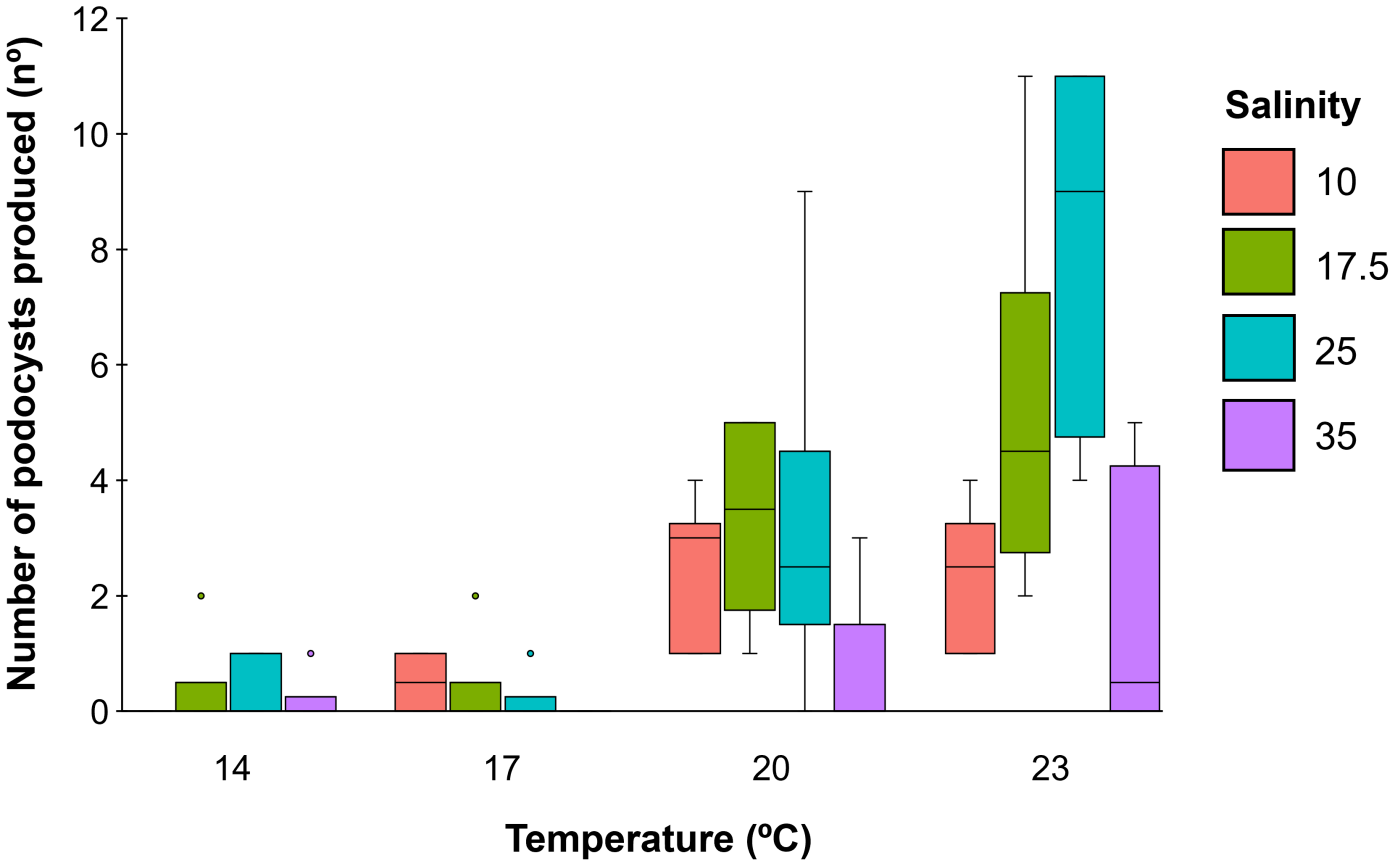
Variation in podocyst production by *Catostylus tagi* polyps under different temperature 14, 17, 20, and 23 °C) and salinity (10, 17.5, 25, and 35) conditions. Values are presented as means ± standard deviation, with *N* = 6 for each temperature and salinity combination.

The podocyst production by polyps exposed to different temperature and salinity conditions is presented in Table S1 and Fig. 6. Both temperature and salinity had significant effects on podocyst production (*p* < 0.05), with a significant interaction between the two factors (*p* < 0.05), being also observed (Tables 1, 2). For the temperature, significant differences among salinities were observed only at the highest temperature (23 °C), where podocyst production was greater at a salinity of 25 compared to salinities of 10 and 35 (Tables S1, S2). Regarding salinity, at 10 and 17.5, podocyst production was significantly higher at elevated temperatures (20 and 23 °C) compared to lower temperatures (14 and 17 °C). At 25, the highest podocyst production occurred at 23 °C. At a salinity of 35, no significant differences were observed among the temperatures tested.

**Table 2:** One-way ANOVA of the effects of temperature and salinity on podocyst production. (-) Not significant. When significant differences are detected for temperature and/or salinity, different lowercase letters are used to denote which specific categories differ significantly from each other (*P* < 0.05).

	Podocyst production															
Temperature (°C)	14				17				20				23			
Salinity	10	17.5	25	35	10	17.5	25	35	10	17.5	25	35	10	17.5	25	35
	–	–	–	–	–	–	–	–	–	–	–	–	a	ab	b	a
																
	Podocyst production															
Salinity	10				17.5				25				35			
Temperature (°C)	14	17	20	23	14	17	20	23	14	17	20	23	14	17	20	23
	a	a	b	b	a	a	b	b	a	a	a	b	–	–	–	–

Most of the podocysts produced by the polyps remained encysted in the wells until the end of the experiment. However, under specific salinity conditions, some podocysts whitened and developed into new polyps (Figs. 5C–5D). Salinity significantly influenced the development of podocysts into new polyps (*p* < 0.01) (Table 1). New polyps were observed in treatments with the lowest salinities (10 and 17.5), while no polyps developed at higher salinities (25 and 35), except for a single podocyst that developed at 25. Statistical analysis confirmed significantly higher polyp development at a salinity of 10 compared to salinities of 25 and 35 (Table S1, Table 1). There were no significant effects of temperature or temperature-salinity interactions on polyp development (Table 1).

### Effects of temperature and salinity on polyp strobilation

**Figure 7:**
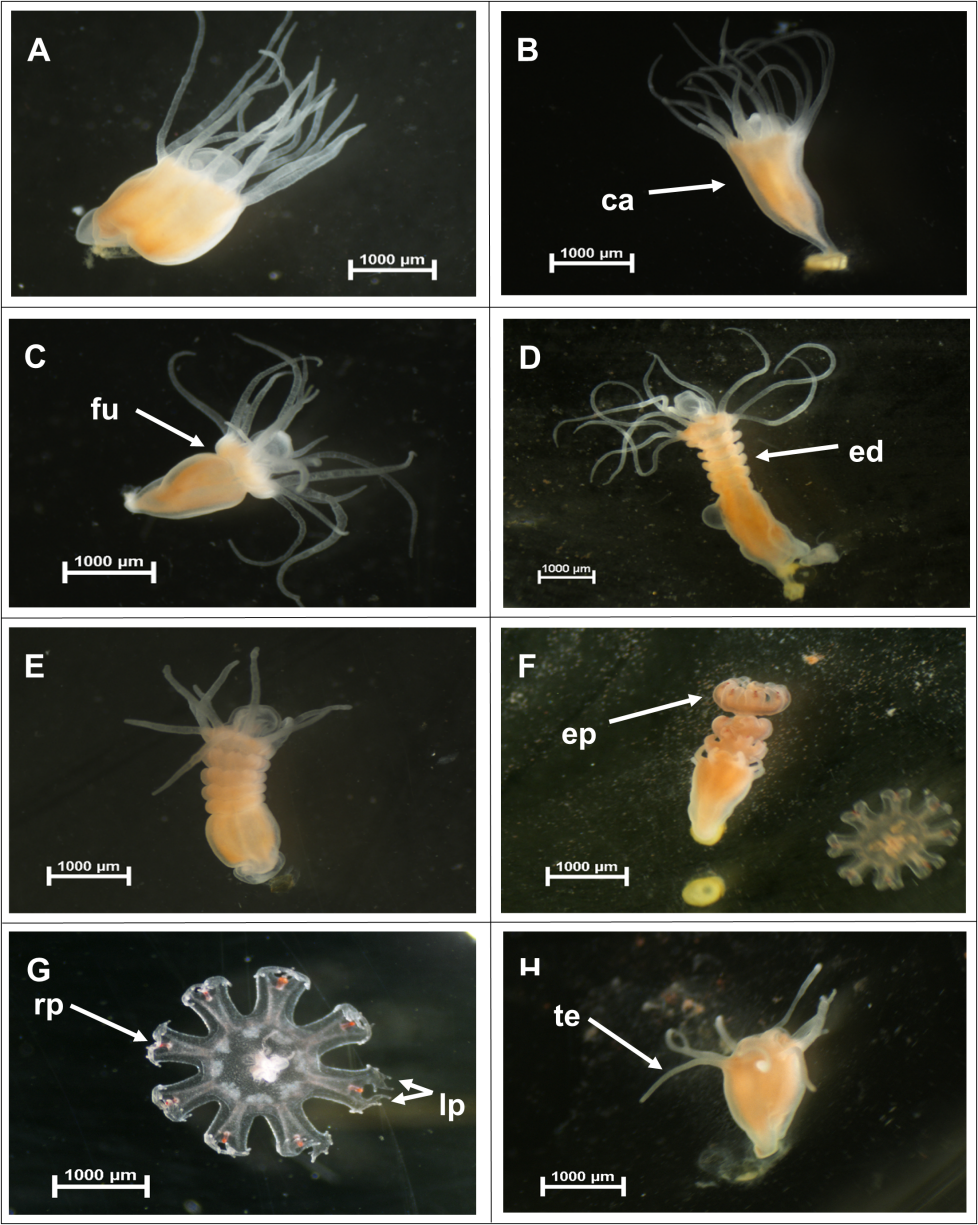
Strobilation cycle of *Catostylus tagi* polyps. (A–H) ca - Calyx; fu - Furrow; ed - Ephyra disc; ep - Ephyra; rp - Rhopalia; lp - Lappets; te - Tentacle. Image credits: Pedro F. Silva.

**Figure 8:**
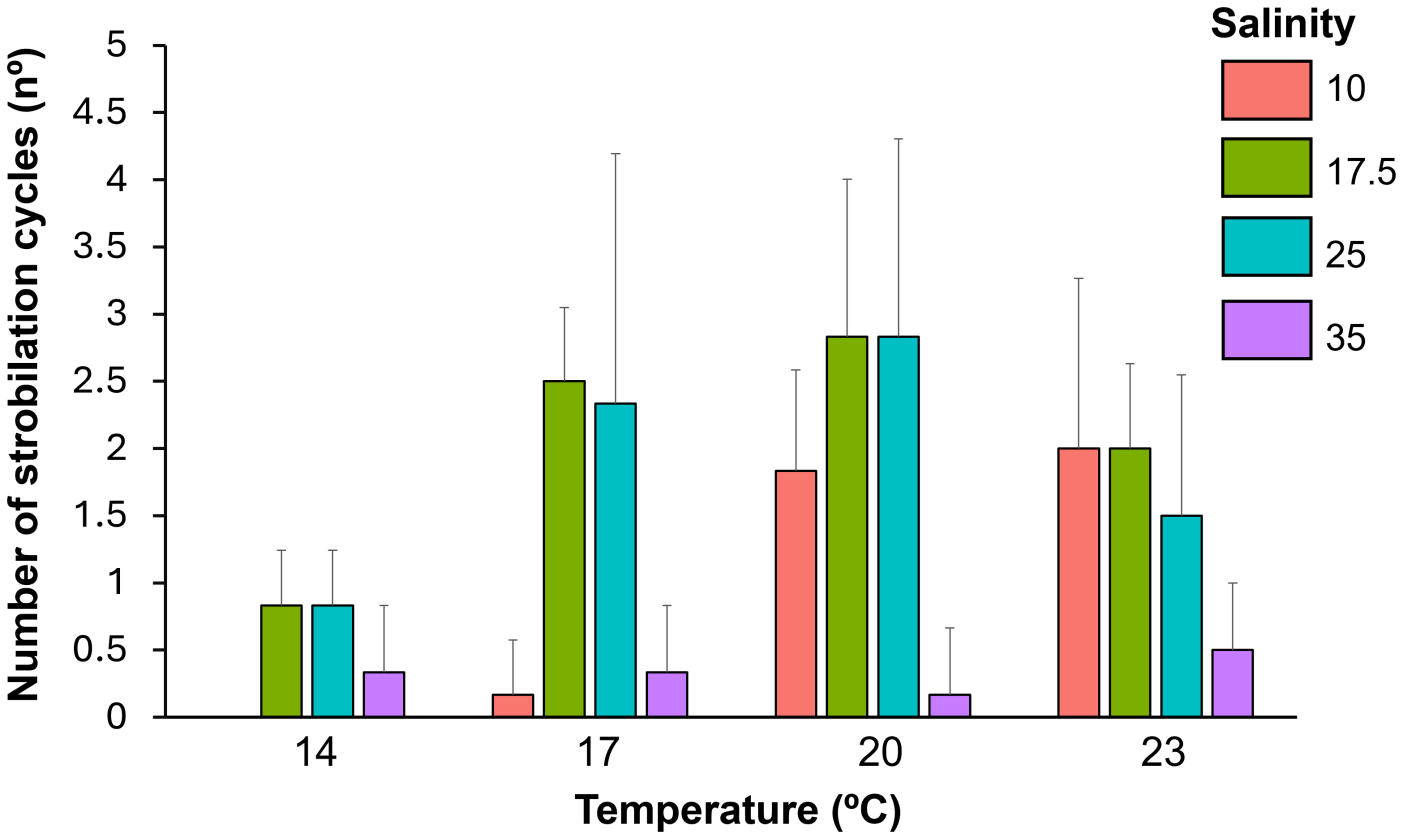
Variation in the number of strobilation cycles performed by *Catostylus tagi* polyps under different temperature (14, 17, 20, and 23 °C) and salinity (10, 17.5, 25, and 35) conditions. Values represent means ± standard deviation, with *N* = 6 for each temperature and salinity combination.

The mature polyps of *C. tagi* exhibited the typical structure of scyphozoan polyps (Fig. 7A), featuring 16 tentacles arranged around the mouth, which are used for food capture. At the onset of the strobilation process, the polyps displayed a noticeable elongation of the calyx (Fig. 7B), allowing an increase in body size that facilitated subsequent ephyrae morphogenesis. This was followed by the formation of a furrow (Fig. 7C), with additional lobes developing sequentially below the initial one (Fig. 7D), culminating in the formation of an ephyra disc. Most polyps exhibited a polydisc strobilation state, producing multiple ephyrae per cycle, while a monodisc state (releasing a single ephyra per cycle) was observed twice in only 2 out of the 96 polyps examined. Upon completion of an ephyra disc, the polyps began to resorb their tentacles (Fig. 7E) as the ephyrae developed rhopalia and lappets (Fig. 7G). Ephyrae initiated pulsation prior to detachment from the parent polyp, and tentacle regeneration in the parent polyp began before the release of all ephyrae (Fig. 7F). After the complete release of the ephyrae, the polyps fully regenerated the tentacles and a functional mouth (Fig. 7H). Following a strobilation cycle, the polyps were notably smaller in size, reflecting the energy expended during ephyrae morphogenesis. The duration of a strobilation cycle varied depending on the tested conditions, ranging from 4 to 25 days. The total number of strobilation cycles during the 71-day experiment at each temperature and salinity is presented in Table S1 and Fig. 8.

Temperature and salinity had significant effects on the number of polyp strobilation cycles (*p* < 0.05), with a significant interaction being also observed between these factors (*p* < 0.05) (Table 1). Regarding temperature, the highest (23 °C) did not significantly influence the number of strobilation cycles observed across the salinity levels tested (Table 3). At 14 °C, a greater number of strobilation cycles was observed at salinities of 17.5 and 25 compared to 10. Similarly, at 17 °C, salinities of 17.5 and 25 resulted in significantly higher numbers of strobilation cycles than salinities of 10 and 35. At 20 °C, salinities of 17.5 and 25 also resulted in a significantly higher number of strobilation cycles compared to 35. Regarding the salinity parameter, at 10, higher temperatures (20 and 23 °C) led to a greater number of strobilation cycles compared to lower ones (14 and 17 °C). At 17.5, temperatures of 17 and 20 °C yielded a significantly higher number of strobilation cycles than 14 °C. For salinities of 25 and 35, no significant differences in the number of strobilation cycles were observed across the temperatures tested.

**Table 3:** One-way ANOVA of the effects of temperature and salinity on the number of strobilation cycles. (-) Not significant. When significant differences are detected for temperature and/or salinity, different lowercase letters are used to denote which specific categories differ significantly from each other (*P* < 0.05).

	Strobilation cycles (n°)															
Temperature (°C)	14				17				20				23			
Salinity	10	17.5	25	35	10	17.5	25	35	10	17.5	25	35	10	17.5	25	35
	a	b	b	ab	a	b	b	a	ab	b	b	a	–	–	–	–
																
	Strobilation cycles (n°)															
Salinity	10				17.5				25				35			
Temperature (°C)	14	17	20	23	14	17	20	23	14	17	20	23	14	17	20	23
	a	a	b	b	a	b	b	ab	–	–	–	–	–	–	–	–

**Figure 9:**
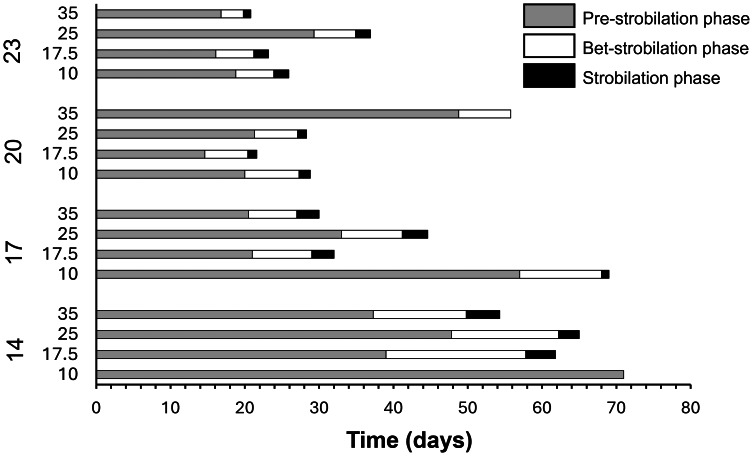
Duration of the pre-strobilation, bet-strobilation, and strobilation phases of *Catostylus tagi* polyps exposed to four temperatures (14, 17, 20, and 23 °C) and four salinities (10, 17.5, 25, and 35) over a 71-day experimental period.

In addition, the duration (in days) of each phase of the strobilation cycle was also examined (Fig. 9). Regarding the pre-strobilation phase, defined as the time the polyps remain in the mature state before initiating their strobilation cycle, both temperature and salinity significantly influenced the duration of this phase (*p* < 0.05) (Table 1). Furthermore, a significant interaction between temperature and salinity was also observed (*p* < 0.05). Regarding temperature, it was observed that the duration of the pre-strobilation phase was not significantly influenced by the temperature of 23 °C across the salinity levels tested (Table 4). At 14 °C, the salinities of 17.5 and 35 resulted in shorter pre-strobilation phases compared to the salinity of 10. Similarly, at 17 °C, the salinity of 10 exhibited the longest pre-strobilation phase among all salinities tested. Conversely, at 20 °C, the longest pre-strobilation phase was recorded at the salinity of 35. Regarding salinity, the pre-strobilation phase was not significantly affected at salinities of 25 and 35 across the tested temperature values. However, for salinities of 10 and 17.5, temperatures of 20 and 23 °C resulted in shorter pre-strobilation periods compared to 14 °C. For the bet-strobilation phase, defined as the time from the onset of strobilation until the release of the first ephyra, both temperature and salinity significantly influenced the duration of this phase (*p* < 0.05), with also a significant interaction between temperature and salinity being observed (*p* < 0.05) (Table 1). Due to the limited number of occurrences in certain groups where polyps underwent the strobilation process, statistical comparisons were only feasible for temperatures of 14, 20, and 23 °C and salinities of 17.5 and 25. Regarding the temperature parameter, the duration of the bet-strobilation phase was not significantly affected by temperatures of 20 and 23 °C across the salinity levels tested. At 14 °C, the salinity of 17.5 exhibited the longest bet-strobilation period compared to 35. No polyps initiated the strobilation process at a salinity of 10. Regarding salinity, at 17.5, the duration of the bet-strobilation phase was significantly longer at lower temperatures (14 and 17 °C). Similarly, at 25, the lowest temperature (14 °C) exhibited a significantly longer bet-strobilation period compared to the higher temperatures. The strobilation phase, defined as the period between the release of the first and last ephyra by the polyps, was significantly influenced only by temperature (Table 1). Polyps at 14 and 17 °C had a longer strobilation period than those at higher temperatures (20 and 23 °C, Table S1). Neither temperature or salinity significantly affected the number of ephyra produced (*p* > 0.05). The duration of the polyps’ first strobilation cycle under the different experimental conditions is shown in Fig. 9.

**Table 4:** One-way ANOVA of the effects of temperature and salinity on the pre-strobilation and bet-strobilation phases. (-) Not significant. When significant differences are detected for temperature and/or salinity, different lowercase letters are used to denote which specific categories differ significantly from each other (*P* < 0.05).


	Pre-strobilation															
Temperature (°C)	14				17				20				23			
Salinity	10	17.5	25	35	10	17.5	25	35	10	17.5	25	35	10	17.5	25	35
	b	a	ab	a	b	a	a	a	a	a	a	b	–	–	–	–
																
	Pre-strobilation															
Salinity	10				17.5				25				35			
Temperature (°C)	14	17	20	23	14	17	20	23	14	17	20	23	14	17	20	23
	b	b	a	a	b	ab	a	a	–	–	–	–	–	–	–	–
					Bet-strobilation							
Temperature (°C)	14				17				20			
Salinity	10	17.5	25	35	10	17.5	25	35	10	17.5	25	35
		b	ab	a	–	–	–		–	–	–	–
			Bet-strobilation					
Salinity	17.5				25			
Temperature (°C)	14	17	20	23	14	17	20	23
	c	b	ab	a	b	a	a	a

### Citizen science and environmental data

**Figure 10:**
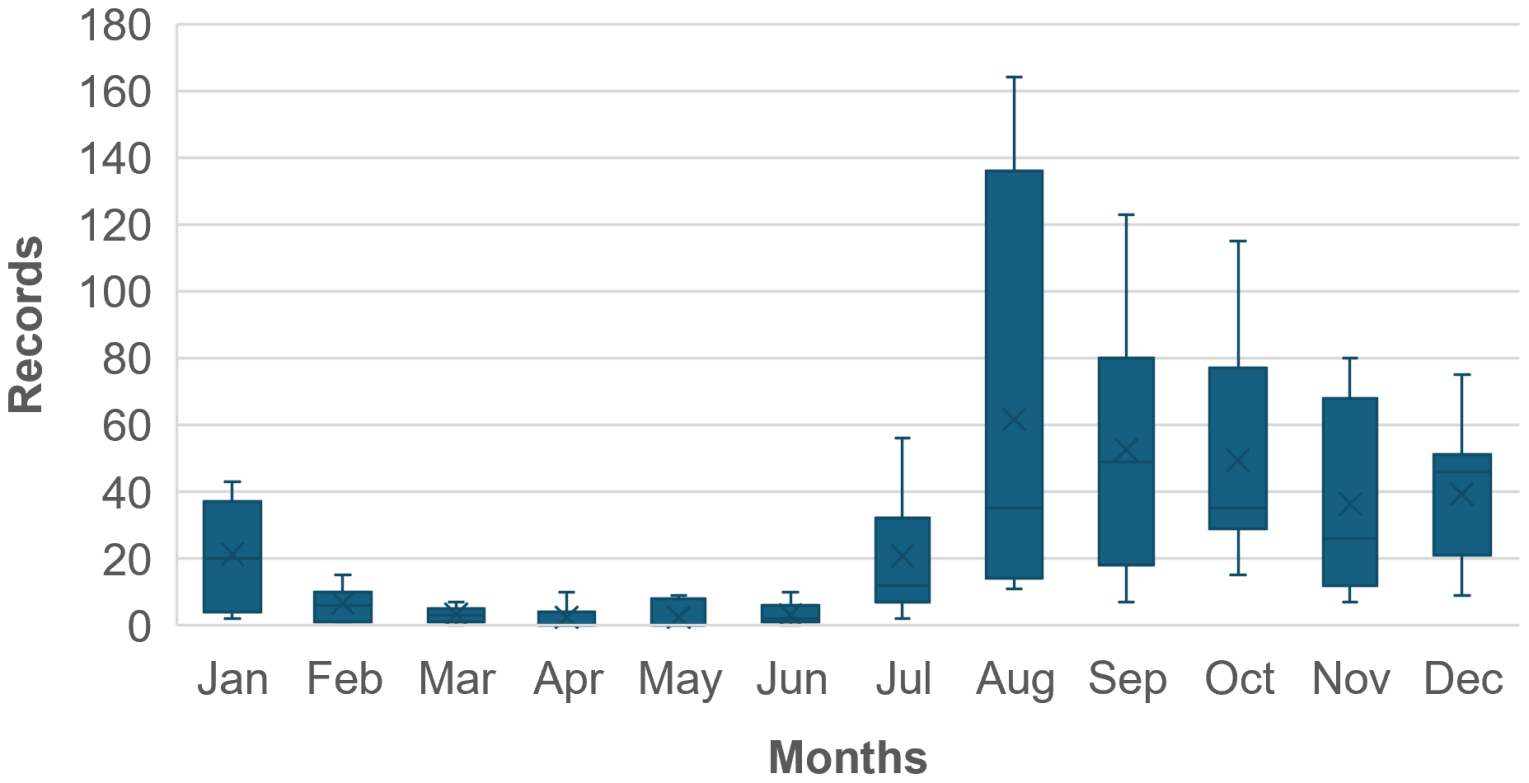
Monthly records of *Catostylus tagi* from the study area, as reported to GelAvista between 2016 and 2022.

Citizen data indicated that the species is present in its medusa phase throughout the Tagus estuary, from area 5 (upstream) to coastal areas, year-round, with the highest frequency of observations occurring between July and January. In contrast, sightings were least common during April, May and June (Fig. 10). The species’ seasonal distribution and abundance are influenced by its life cycle, as well as by citizen density within the study area and the local topography. Observation numbers tended to be lower in salt marshes and regions with limited water access, such as zones 7 (Lower South Bank) and 6 (Upper South Bank), while higher counts were recorded in areas featuring riverside promenades or beaches, including zones 3 (Lower North Bank), 2, and 8 (Costa da Caparica beaches). Considering the citizen-data analysis, several environmental variables were shown to be significantly correlated with *C. tagi* abundance (Table 5): Sea surface temperature, wind speed and wind speed separated by quadrants. In fact, wind speed negatively affected the abundance of *C. tagi* when considering all zones together (*R* = −0.290, *p* < 0.001; Rs = −0.318, *p* < 0.001). Furthermore, SST also showed a positive and significant influence on sightings abundances (*R* = 0.095, *p* = 0.021; Rs = 0.115, *p* = 0.005).

**Table 5:** Correlation between environmental variables and *Catostylus tagi* abundance (log transformed) in the Tagus estuary and adjacent areas and separated by areas. Values presented correspond to Pearson correlations followed by Spearman correlations, between brackets.

				Area				
	All areas	1	2	3	4	6	7	8
Precipitation	0.073 [0.025]	0.043 [-0.042]	−0.027 [−0.120]	0.085 [0.016]	−0.050 [−0.059 ]	0.180 [0.122]	0.088 [0.026]	0.027 [0.031]
Air temperature	−0.009 [−0.014]	0.086 [0.052]	0.106 [0.099]	−0.097 [−0.080]	0.180 [0.186]	−0.065 [−0.058]	−0.096 [−0.116]	−0.230 [−0.162]
Chlorophyll a	−0.020 [0.006]	−0.107 [0.011]	0.015 [−0.009]	−0.073 [−0.037]	0.100 [0.073]	0.036 [0.055]	−0.232^*^ [−0.172]	−0.098 [−0.104]
SST	0.095^*^ [0.115^*^]	0.212 [0.242^*^]	0.272^*^ [0.301^*^]	0.006 [0.026]	0.175 [0.191]	0.184 [0.185]	0.245^*^ [0.232^*^]	0.097 [0.101]
Wind direction	0.043 [0.046]	−0.074 [0.00]	−0.030 [−0.062]	0.087 [0.065]	0.119 [0.140]	0.238 [0.180]	0.215 [0.167]	−0.035 [−0.042]
Wind speed	−0.290^*^ [−0.318^*^]	0.120 [0.071]	−0.189 [0.189]	−0.161 [−0.151]	0.160 [0.150]	−0.293^*^ [−0.283^*^]	−0.242 [−0.265^*^]	−0.119 [−0.118]
N-W	0.310^*^ [−0.358^*^]	0.047 [0.054]	−0.150 [−0.156]	−0.187 [−0.187]	0.134 [0.135]	−0.318^*^ [−0.324^*^]	−0.226 [−0.243]	−0.096 [−0.062]
S-W	−0.375^*^ [−0.341^*^]	−0.639 [−0.500]	−0.533 [−0.543]	−0.018 [0.134]	No cases	−0.085 [0.000]	−0.719 [−0.707]	0.054 [−0.355]
N-E	0.091 [0.061]	0.979^*^ [0.872]	−0.012 [0.229]	0.149 [0.411]	0.604 [0.707]	0.640 [0.616]	0.441 [0.359]	−0.350 [−0.439]
S-E	−1^*^ [−1^*^]	No cases	No cases	No cases	No cases	No cases	No cases	No cases

**Notes.**

Significant values are marked with *

The cross-correlation analysis between *C. tagi* abundance and sea surface temperature, presented in Table 6, shows that the correlation values between variables are much increased when the temperature lags two or three months earlier.

**Table 6:** Pearson correlations between *Catostylus tagi* abundance (log transformed) in the Tagus estuary and adjacent areas and separated by areas (see text) with SST lagged 0 to 5 months earlier.

Lagged SST					Area			
	All areas	1	2	3	4	6	7	8
−5	0.078	0.199	0.006	0.277^*^	0.140	0.051	0.081	0.079
−4	0.258^*^	0.546^*^	0.305^*^	0.479^*^	0.353^*^	0.137	0.344^*^	0.171
−3	0.345^*^	0.688^*^	0.493^*^	0.512^*^	0.483^*^	0.347^*^	0.515^*^	0.101
−2	0.332^*^	0.692^*^	0.449^*^	0.430^*^	0.471^*^	0.278^*^	0.574^*^	0.127
−1	0.234^*^	0.501^*^	0.404^*^	0.257^*^	0.379^*^	0.279^*^	0.394^*^	0.047
0	0.095^*^	0.212	0.272^*^	0.06	0.175	0.184	0.245^*^	0.097

**Notes.**

Significant values presented with *.

Moreover, the different generalized estimation equation models adjustments, presented in Table S2, showed that the best fit model, with the lowest QIC, included sea surface temperature two and three months earlier (SST2 and SST3), and wind speed as predictor variables to explain the occurrence of *C. tagi* in the studied area. The best-fit model showed that wind speed and sea surface temperatures have a significant impact on the probability of medusae occurrence (Table S3). Wind intensity was negatively correlated, meaning that lower wind intensity increased the likelihood of jellyfish observations. On the other hand, sea surface temperatures from two and three months prior to the recorded abundance of *C. tagi* had a positive effect, suggesting that higher temperatures during the ephyrae stage were associated with a greater probability of jellyfish observations.

## Discussion

Polyps survived in nearly all experimental treatments, except those exposed to a salinity of 35, where high mortality rates were observed. In contrast, no mortality occurred at the other salinity levels, and no significant differences on survival were detected between the tested temperatures. Although no mortality was recorded at 14 °C, polyps in this condition exhibited reduced mobility and activity compared to those maintained at higher temperatures, suggesting that 14 °C may not be optimal for their development and growth or that a longer observation period may be required to detect potential effects. These findings emphasize their adaptability to varying environmental conditions and the estuarine nature of the species. Although *C. tagi* polyps have not yet been observed in nature, and medusae of this species are commonly sighted along the Tagus estuary and in adjacent coastal waters, the results of this study suggest that salinities below 35 are more favorable for polyp survival and development. Consequently, it is likely that the polyps may be in the upper estuary of the Tagus river, where salinity levels align with those tested in this study (Rodrigues et al., 2017) and that few polyps will develop at the coastal areas. Moreover, the medusae found in the lower estuary and coastal areas must have an estuarine origin and be displaced by tide and current to the ocean area. According to the present results, salinity is likely to be a key parameter preventing the asexual reproduction in the ocean, with implications for the population expansion into new areas. However, the present findings confirm the eurythermal and euryhaline nature of *C. tagi* polyps, as they demonstrated survival across most tested temperature and salinity ranges. These traits, previously observed in *C. tagi* planulae, are consistent with the thermal and salinity tolerance exhibited by planulae and polyps of many scyphozoan species inhabiting estuarine environments, including *Chrysaora pacifica*, *Aurelia coerulea*, *Nemopilema nomurai*, and *Rhopilema esculentum* (Conley & Uye, 2015; Dong et al., 2015; Gueroun et al., 2021; Takao & Uye, 2018).

In contrast to *Catostylus mosaicus*, podocyst production was identified as the sole propagation strategy in *C. tagi* polyps, corroborating the findings of Gueroun et al. (2021). In the present study, *C. tagi* polyps, which were fed twice a week, produced podocysts on the substrate *via* their attached pedal discs, a mechanism that had been observed in most species, including *Rhopilema nomadica* (Lotan, Ben-Hillel & Loya, 1992). Once a podocyst was formed on the pedal disc, the polyp could produce a stolon to relocate, leaving the podocyst behind. At the new location, the polyp could establish another pedal disc and potentially form additional podocysts, creating a chain-like cascade of podocysts. In contrast, *C. mosaicus* formed podocysts directly on the substrate through stolons, bypassing the involvement of pedal discs (Pitt, 2000).

Previous research has challenged the earlier assumption that podocysts are produced exclusively under adverse conditions. In fact, studies have shown that podocyst production rates are generally positively correlated with food availability. For example, Guo (1990) demonstrated that *Rhopilema esculenta* polyps fed daily with *Artemia* nauplii produced more podocysts, and had a higher rate of excystment, compared to polyps when the daily feeding was interrupted, which resulted in reduced podocyst production and lower excystment success. In contrast, Gueroun et al. (2021) reported that *C. tagi* podocyst production occurred independently of the feeding regime, as podocysts were formed even when polyps were not fed. However, these podocysts did not excyst, suggesting that while podocyst formation may occur regardless of food availability, their development and successful excystment may still depend on other factors.

In the present study, podocyst production by *C. tagi* was found to be influenced by temperature, contradicting the findings of Gueroun et al. (2021) on the same species, who reported no significant effect of temperature on podocyst production. This discrepancy may be attributed to the longer experimental period of our study (71 days), compared to the shorter duration (33 days) in Gueroun et al. (2021), as well as the inclusion of salinity as an additional factor. In the present study, a significantly higher production of podocysts in *C. tagi* was observed at higher temperatures (20 and 23 °C), while lower temperatures (14 and 17 °C) resulted in minimal podocyst production. These findings suggest that temperature is a critical factor influencing the rate of podocyst production in *C. tagi*, particularly that it relies solely on this asexual reproduction type strategy. Similarly, in *Rhizostoma luteum* and *Nemopilema nomurai*, an increase in temperature has been shown to enhance podocyst production rates (Feng et al., 2015; Kienberger et al., 2018). Conversely, in species with multiple asexual reproduction strategies, temperature has been noted to promote alternative methods such as gemmulation.

Podocyst production was higher at elevated temperatures (20 and 23 °C), particularly at intermediate salinities (17.5 and 25). Additionally, lower salinities (10 and 17.5) were found to be more conducive to the development of podocysts into new polyps, irrespective of temperature. In contrast, podocysts formed at the highest salinities (25 and 35) generally remained encysted throughout the experimental period. The sole exception was a single podocyst at 25, which successfully developed into a polyp. These findings reinforce the hypothesis that *C. tagi* polyps likely favor upstream areas of the Tagus estuary, where salinity levels are more suitable for podocyst production and subsequent polyp multiplication. This supports the adaptability of the species to estuarine environments and highlights the critical role of salinity in influencing their asexual reproduction dynamics. It can be inferred that the rise in temperature during the spring months promotes increased podocyst production by *C. tagi* polyps. In areas with lower salinity levels (10–17.5), these podocysts develop into new polyps, significantly contributing to the asexual reproduction and efficient propagation of the species. Podocysts play a vital role in ensuring the survival of *C. tagi* polyp populations during the colder months of December and January, when water temperatures can drop to 11 °C as in February of 2018 and January of 2021 (Rodrigues et al., 2017). Citizen science data indicates that *C. tagi* sightings during those two years were notably lower than in other years, with this trend being especially pronounced in the spring months. This mechanism is particularly important because decreasing temperatures during winter can pose significant risks to polyp survival. Podocysts appear to act as a survival strategy, preserving population viability throughout the winter months. Further studies are needed to investigate the relevance of water dynamics in this process.

Temperature and salinity play a critical role in regulating the number of strobilation cycles in *C. tagi*, with lower temperatures potentially constraining strobilation frequency, while higher temperatures may promote a more recurrent reproductive process. In other scyphozoans, environmental conditions also strongly influence strobilation dynamics, although the specific temperature response can differ among species. For instance, polyps of *Chrysaora hysoscella* and *Aurelia aurita* initiated strobilation when water temperature decreased from 15 °C to 10 °C during autumn (Holst, 2012), whereas *Rhizostoma pulmo* exhibited a higher frequency of strobilation cycles at temperatures between 21 °C and 28 °C (Purcell et al., 2012). While strobilation can still occur at lower temperatures, suboptimal conditions may compromise ephyrae development, as evidenced by mobility impairments and morphological deformities. These findings suggest that cooler temperatures could impose physiological constraints on reproductive success and ephyrae viability in *C. tagi*. Moreover, the observed relationship between temperature and strobilation frequency provides valuable insights into how this process can be controlled in *ex situ* conditions. Lower temperatures could be used strategically to suppress strobilation, which is particularly relevant for optimizing culturing practices, managing resource allocation, and preserving polyp stocks. Further research is needed to refine these strategies and better understand the mechanisms regulating strobilation dynamics in controlled environments.

Salinity plays a significant role in regulating strobilation in *C. tagi*, with intermediate salinity levels (*e.g.*, 17.5 and 25) generally promoting a higher frequency of strobilation cycles. This suggests that salinity may influence reproductive success by altering physiological conditions that affect energy allocation for reproduction. In contrast, higher salinities, such as 35, appear to suppress strobilation, potentially due to increased energy expenditure on osmoregulation. At low salinities, such as 10, strobilation occurred predominantly at higher temperatures (20–23 °C), which could indicate that salinity and temperature interact to shape the strobilation process. These findings highlight the complexity of the relationship between salinity and strobilation, suggesting that certain salinity levels may disrupt or limit reproductive processes in *C. tagi*. Similar patterns have been observed in other scyphozoan species, where low salinities delayed or reduced strobilation, likely by increasing the energetic demands of osmoregulation (Purcell, Hoover & Schwarck, 2009).

In scyphozoan polyps, energy resources must be allocated not only between asexual reproduction and strobilation, but also among survival, growth, and other physiological processes. This allocation reflects the finite energy available to all animals and can be interpreted within the framework of the Dynamic Energy Budget theory (Sousa et al., 2010). Therefore, environmental conditions, specifically temperature and salinity, that support both processes are likely to optimize population growth (Purcell et al., 2012). At 23 °C, it was observed that while the polyps were producing podocysts, they remained in the pre-strobilation phase, and conversely, when undergoing strobilation, podocyst production ceased. This alternation between strobilation and podocyst production highlights the dynamic energy trade-offs in response to environmental conditions. At a salinity of 10, a greater number of strobilation cycles were observed compared to salinities of 17.5 and 25 at 23 °C. However, podocyst production was lower under these conditions, suggesting that the polyps allocated more energy toward the strobilation process. Consistent with observations in other Rhizostomatoidea species, except for *Rhizostoma luteum*, *C. tagi* exhibited polydisc strobilation, characterized by the release of multiple ephyrae per strobilation cycle (Gueroun et al., 2021).

Regarding the strobilation cycles, temperature and salinity not only influenced the number of cycles but also affected their duration. Higher temperatures (20 °C and 23 °C) shortened the strobilation cycles of *C. tagi* compared to lower temperatures (14 °C and 17 °C), which delayed the onset and extended the duration of the cycles. These findings align with those of Gueroun et al. (2021), who reported that *C. tagi* strobilation cycles were shorter at 20 °C than at 15 °C. Temperature has been shown to play a critical role in modulating the duration of strobilation cycles in various scyphozoan polyps. For example, in *Rhizostoma pulmo*, polyps released their first ephyrae more rapidly at 28 °C compared to 21 °C (Purcell et al., 2012). Notably, at 17.5 salinity, the pre-strobilation period was shorter compared to other salinity levels.

This adaptability likely plays a crucial role in regulating the distribution and dispersal of *C. tagi* in their natural habitats. In this study, the number of ephyrae produced per strobilation cycle was not significantly affected by the tested temperature and salinity conditions. This suggests that within the specific range of temperatures and salinities examined, these environmental factors did not elicit notable differences in ephyrae production. However, it is important to note that variations in ephyrae production may still occur outside the tested ranges, and further studies incorporating additional factors, such as food availability, polyp size, or other environmental variables, are needed to validate the findings observed in this study. Such factors have been shown in previous research to significantly influence ephyrae production. For example, Purcell et al. (1999) and Russell (1970) found that both temperature and food availability could affect the number of ephyrae produced by polyps.

In the Tagus estuary, *C. tagi* medusae are observed throughout the year, with reduced abundance during the spring months (April to June), which is probably related to the lower strobilation during winter, likely accompanied by reduced survival rates of ephyrae. Consistent with this, the present study observed a reduction in the number of strobilation cycles at lower temperatures compared to higher temperatures. However, strobilation was not entirely absent, consistent with the seasonal occurrence of medusae throughout the year in the natural environment, except during spring months when they are rarely seen. These findings indicate that while the strobilation process is diminished at lower temperatures, certain polyps and ephyrae are capable of surviving, sustaining the population through less favorable conditions. As temperatures begin to rise during the spring months, polyps might proliferate and initiate the strobilation process, particularly in areas where salinity ranges from 10 to 25.

The analysis of the *C. tagi* sightings data provided by the GelAvista project showed that the adult stage occurs throughout the year, in all areas of the Tagus estuary and adjacent coastal areas. Although limited to the adult population dynamics, our analyses showed that the abundance of the medusae is related to the wind speed and to water temperature 2 to 4 months earlier than observations. These results appear to align with the reproduction and growth phases of *C. tagi*. Juveniles of this species require approximately 5 months for full development (Gueroun et al., 2021). Therefore, the period of 2, 3, and 4 months may represent the time needed for the medusae to grow and reach a size that makes them visible to observers. In fact, the integration of our laboratory and field results suggest that the polyps of this species might occur in the upper Tagus estuary, where salinities are more favourable, with higher podocyst production and strobilation cycles at intermediate temperatures and salinities (17–20 °C and 17.5–25), and ephyrae may then disperse to the areas of the lower estuary and adjacent coastal areas due to estuarine currents, reaching them 2 to 4 months later.

Wind was identified as a key factor influencing the distribution and abundance of *C. tagi* throughout the study area. The significant negative impact of wind direction suggests that, as anticipated, this jellyfish has difficulty counteracting the force of surface currents, which are largely governed by wind (Peliz et al., 2002). Additionally, north westerly winds, prevalent along the Portuguese coastline (Peliz et al., 2005) and sometimes strong, tend to drive individuals away from the estuary and coastal zones, leading to reduced observation rates. Furthermore, the negative correlations found for zones 2 (Lower North Bank) and 3 (Coastal north area) with winds from the NW and NE quadrants align with evidence of the medusae limited capacity to resist currents; these wind patterns displace them from the shoreline in these areas, resulting in fewer recorded sightings.

Although citizen science data is inherently limited, it remains valuable for elucidating patterns in jellyfish distribution and abundance and for validating laboratory findings. An enhancement to this methodology could include organizing sampling sites with active citizen involvement, thereby supplementing collected data in a targeted and comprehensive manner. These initiatives may foster a deeper understanding of species ecology and underscore the essential contribution of citizen science to acquiring critical information needed for marine conservation and sustainable management. Additionally, integrating the coastal upwelling index as a variable of interest would facilitate a more nuanced analysis of wind-related and oceanographic influences on species distribution and abundance. Advanced knowledge of *C. tagi* ecology is fundamental to the development of effective management and conservation strategies for coastal ecosystems inhabited by this species (Stratoudakis et al., 2022).

Overall, this integrative approach connects experimental and citizen science data, demonstrating how environmental factors regulate both the early and adult stages of *C. tagi*. Laboratory findings identified optimal temperature and salinity ranges for survival and reproduction, while field observations confirmed that temperature at time of ephyrae development corresponds to periods and locations of higher medusae abundance and that wind intensity is a main driver of its abundance. Together, these results provide a unified understanding of the species’ life cycle, showing that temperature and salinity are key drivers shaping the temporal and spatial dynamics of *C. tagi* populations in the Tagus estuary and adjacent coastal waters.

## Conclusions

The species *Catostylus tagi* is found in Portuguese coastal areas, especially in the Tagus and Sado estuaries, and medusae can be observed throughout the year, most frequently from July to January. Understanding how environmental factors, such as seawater temperature, salinity and wind, affect its abundance in its different life stages, including the polyp and the medusa stage, is essential to predict its distribution patterns. The present study demonstrates a high tolerance of *Catostylus tagi* polyps to varying environmental conditions, particularly temperature and salinity. These findings contribute to a deeper understanding of the biology and ecology of this species, addressing critical knowledge gaps regarding the polyp stage. The present findings align with the temporal and spatial distribution of this species in the Tagus estuary, offer significant advantages for future research, and production of *C. tagi* and emphasize the critical role of environmental factors, such as temperature and salinity, in the survival, asexual reproduction, and polyp strobilation. The salinity and temperature ranges identified as most favorable for the survival, asexual reproduction, and strobilation of *C. tagi* polyps are 17.5–25 and 17–20 °C, respectively. By combining citizen science data with environmental parameters, we found that wind exerts a significant influence on the dispersal of this species’ medusae, pushing them away from coastal areas during strong winds. Sea surface temperature, measured 2 to 4 months prior to observations, also affects species abundance, reflecting their life cycle. Nonetheless, further research is essential to explore additional environmental parameters which may also influence the biological processes, such as the inclusion of upwelling indices as part of the investigated variables for a more complete understanding of the underlying coastal processes.

This study also provides an initial understanding of the mechanisms that trigger strobilation, underscoring the importance of identifying additional factors to better comprehend and control this process. It also contributes to our understanding of how temperature and salinity influence asexual reproduction and polyp multiplication. Furthermore, the ephyra stage remains insufficiently studied, and it is crucial to identify the critical environmental factors that impact its survival and growth, as this stage plays a decisive role in the occurrence and abundance of adult medusae, thus contributing to a more complete understanding of the life cycle of *C. tagi*. Overall, studies like the present one are of significant value and are essential for evaluating the response of *C. tagi* to climate change, which appears to promote the dispersal of this species. Additionally, such research is vital for predicting future temporal and spatial distribution of *C. tagi*. Finally, it also demonstrates the high value of citizen science data to add analysis and increase knowledge in multidisciplinary studies.

## Supplemental Information

10.7717/peerj.20862/supp-1 Supplemental Information 1 Coastal and Tagus estuary divided into 8 areas represented by the polygons defined in the legend Area 1- Purple; Area 2- yellow; Area 3- light green; Area 4- light blue; Area 5- green; Area 6- pink; Area 7- orange; Area 8- blue. Image credits: Made by Antonina dos Santos in Ocean Data View Software (Schlitzer,2025).

10.7717/peerj.20862/supp-2 Supplemental Information 2 Average survival (% and time), number of podocysts (produced and developed), and strobilation cycles and phases of *C. tagi* polyps across temperatures and salinities SD –Standard deviation.

10.7717/peerj.20862/supp-3 Supplemental Information 3 Adjustment measure (QIC) of several Generalized Estimation Equation (GEE) models with Catostylus tagi as dependent variable Included independent variables: SST –sea surface temperature; SST1 –sea surface temperature lagged one month; SST2 –sea surface temperature lagged two months; SST3 –sea surface temperature lagged three months; SST4 –sea surface temperature lagged four months; Air temperature; Precipitation; Chlorophyll a; Wind direction and wind speed.

10.7717/peerj.20862/supp-4 Supplemental Information 4 Results from the generalized estimating equation (GEE) model selected.

10.7717/peerj.20862/supp-5 Supplemental Information 5 *C. tagi* Bet-strobilation dataset across temperature and salinity trials

10.7717/peerj.20862/supp-6 Supplemental Information 6 *C. tagi* ephyrae number dataset across temperature and salinity trials

10.7717/peerj.20862/supp-7 Supplemental Information 7 *C. tagi* Pre-strobilation dataset across temperature and salinity trials

10.7717/peerj.20862/supp-8 Supplemental Information 8 *C. tagi* strobilation cycles, survival and podocyst production dataset across temperature and salinity trials

10.7717/peerj.20862/supp-9 Supplemental Information 9 *C. tagi* abundance in monthly intervals collected by citizen scientists from the GelAvista project (2016 to 2022)

## Additional Information and Declarations

### Data Availability

The following information was supplied regarding data availability: The raw data is available in the Supplemental Files.
