# Supplementary figures and images for: Environmental drivers of Catostylus tagi polyp survival and reproduction: unlocking the role of temperature and salinity, supported with citizen science data
Source: PeerJ. 2026 Mar 17;14:e20862. doi: 10.7717/peerj.20862 (PMC13003947; doi:10.7717/peerj.20862)

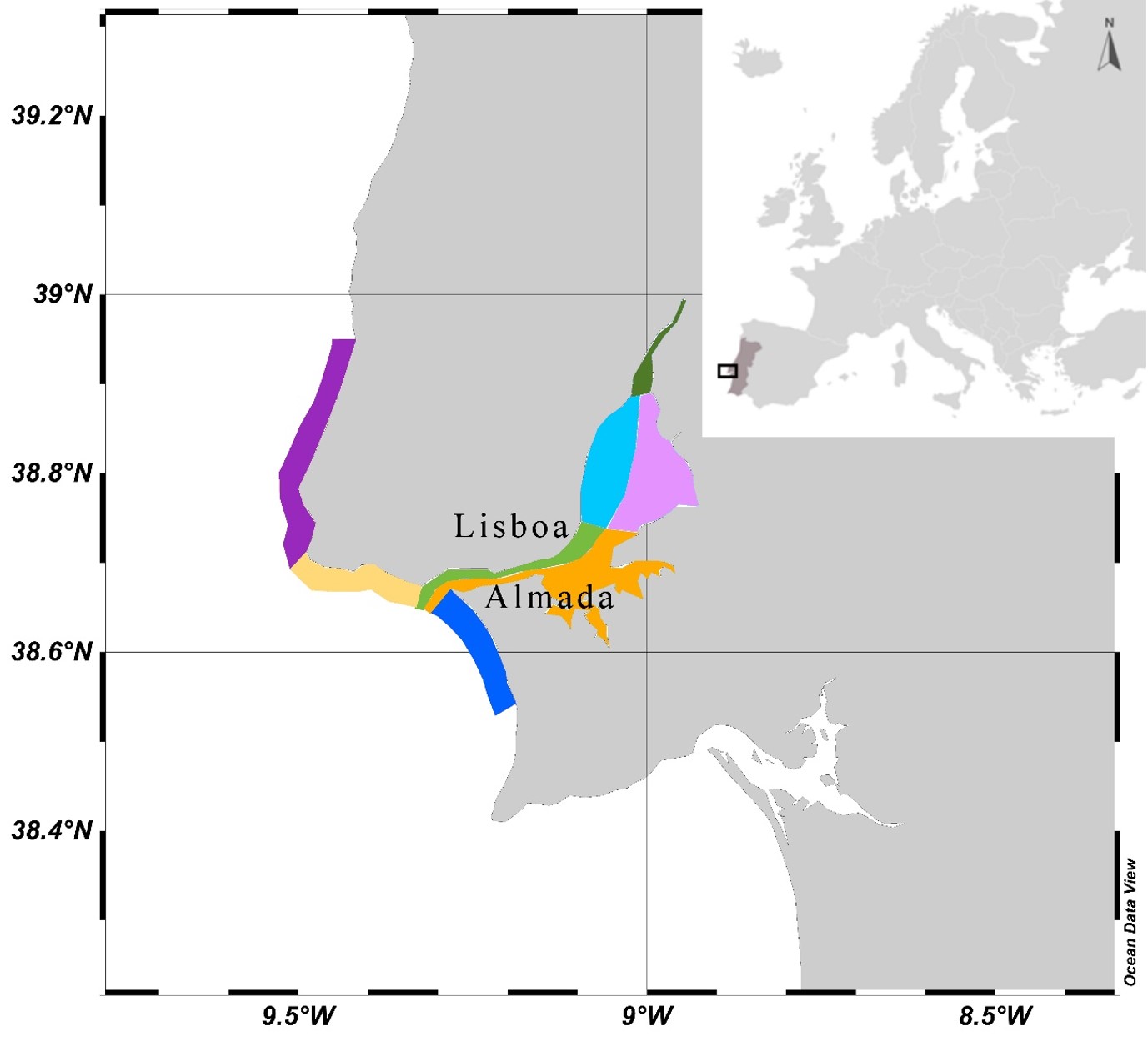

Supplement: Supplemental Information 1 — Area 1- Purple; Area 2- yellow; Area 3- light green; Area 4- light blue; Area 5- green; Area 6- pink; Area 7- orange; Area 8- blue. Image credits: Made by Antonina dos Santos in Ocean Data View Software (Schlitzer,2025). [file peerj-14-20862-s001.jpg]
