# Supplementary material for: Environmental drivers of Catostylus tagi polyp survival and reproduction: unlocking the role of temperature and salinity, supported with citizen science data
Source: PeerJ. 2026 Mar 17;14:e20862. doi: 10.7717/peerj.20862 (PMC13003947; doi:10.7717/peerj.20862)
Supplement: Supplemental Information 2 — SD –Standard deviation. [file peerj-14-20862-s002.docx]

**Table S1: Average survival (% and time), number of podocysts (produced and developed), and strobilation cycles and phases of *C. tagi* polyps across temperatures and salinities (SD – Standard deviation).**

| Salinity |  |  | Temperature (ºC) | |  |  |  |  |
| --- | --- | --- | --- | --- | --- | --- | --- | --- |
|  |  | 14 | 17 | 20 | | | 23 | |
|  |  |  |  |  | | |  | |
| **Survival (%)** |  |  |  |  | | |  | |
| 10 |  | 100 | 100 | 100 | | | 100 | |
| 17.5 |  | 100 | 100 | 100 | | | 100 | |
| 25 |  | 100 | 100 | 100 | | | 100 | |
| 35 |  | 50 | 16.6 | 33.3 | | | 33.3 | |
| **Average survival time in days (SD)** |  |  |  |  | | |  | |
| 10 |  | 71 | 71 | 71 | | 71 | |  |
| 17.5 |  | 71 | 71 | 71 | | | 71 | |
| 25 |  | 71 | 71 | 71 | | | 71 | |
| 35 |  | 43.6 (31.1) | 26 (23.5) | 50 (19.3) | | | 28.5 (32.9) | |
| **Average number of podocysts produced (SD)** |  |  |  |  | | |  | |
| 10 |  | 0 | 0.6 (0.8) | 2.6 (1.0) | | | 2.3 (1.2) | |
| 17.5 |  | 0.3 (0.8) | 0.3 (0.8) | 4.3 (3.2) | | | 5.1 (3.1) | |
| 25 |  | 0.3 (0.5) | 0.1 (0.4) | 3 (3.0) | | | 8.1 (3.0) | |
| 35 |  | 0.1 (0.4) | 0 | 0.6 (1.2) | | | 1.5 (2.3) | |
| **Average number of developed podocysts (SD)** |  |  |  |  | | |  | |
| 10 |  | 0 | 0.5 (0.8) | 1 (0.6) | | | 0.1 (0.4) | |
| 17.5 |  | 0.1 (0.4) | 0 | 0.5 (1.2) | | | 0.5 (0.4) | |
| 25 |  | 0.1 (0.4) | 0 | 0 | | | 0 | |
| 35 |  | 0 | 0 | 0 | | | 0 | |
| **Average number of strobilation cycles (SD)** |  |  |  |  | | |  | |
| 10 |  | 0 | 0.1 (0.4) | 1.8 (0.7) | | | 2 (1.2) | |
| 17.5 |  | 0.8 (0.4) | 2.5 (0.5) | 2.8 (1.1) | | | 2 (0.6) | |
| 25 |  | 0.8 (0.4) | 2.3 (1.8) | 2.6 (1.5) | | | 1.5 (1.0) | |
| 35 |  | 0.3 (0.5) | 0.3 (0.5) | 0.1 (0.4) | | | 0.5 (0.8) | |
| **Strobilation phases** |  |  |  |  | | |  | |
| **Average number of days in pre-strobilation phase (SD)** | | |  |  | | |  | |
| 10 |  | 71 (0) | 63.5 (18.3) | 20 (6.7) | | | 18.8 (20.3) | |
| 17.5 |  | 39 (16.8) | 21 (6.5) | 14.6 (8.8) | | | 16.1 (12.8) | |
| 25 |  | 47.8 (12.6) | 33 (27.7) | 21.3 (24.6) | | | 29.3 (28.6) | |
| 35 |  | 37.3 (25.7) | 20.5 (14.7) | 48.8 (20.4) | | | 16.8 (26.5) | |
| **Average number of days in bet-strobilation phase (SD)** | | |  |  | | |  | |
| 10 |  | 0 | 11 (0) | 7.3 (1.3) | | | 5.1 (0.7) | |
| 17.5 |  | 18.8 (3.0) | 8 (1.7) | 6.1 (2.6) | | | 4.1 (0.9) | |
| 25 |  | 14.4 (2.3) | 8.2 (2.7) | 5.8 (1.0) | | | 5.6 (2.0) | |
| 35 |  | 12.5 (2.12) | 6.5 (2.1) | 7 (0) | | | 3 (0) | |
| **Average number of days in strobilation phase (SD)** | | |  |  | | |  | |
| 10 |  | 0 | 1 | 1.5 (0.5) | | | 2 (0.8) | |
| 17.5 |  | 3.3 (2.3) | 3 (1.0) | 1.8 (1.1) | | | 1.5 (0.8) | |
| 25 |  | 2.8 (0.8) | 3.4 (2.3) | 1.2 (0.4) | | | 2 (1.4) | |
| 35 |  | 4.5 (2.1) | 3 (0) | 0 | | | 1 | |
| **Average number of ephyrae released (SD)** |  |  |  |  | | |  | |
| 10 |  | 0 | 2 | 3.3 (1.3) | | | 4 (1.4) | |
| 17.5 |  | 4.8 (1.6) | 3.6 (1.3) | 4.55 (2.2) | | | 5 (2.2) | |
| 25 |  | 4.2 (1.6) | 5 (4.1) | 3.8 (2.4) | | | 7.4 (3.7) | |
| 35 |  | 2.5 (0.7) | 4.5 (0.7) | 0 | | | 2 | |
