# Supplementary material for: Environmental drivers of Catostylus tagi polyp survival and reproduction: unlocking the role of temperature and salinity, supported with citizen science data
Source: PeerJ. 2026 Mar 17;14:e20862. doi: 10.7717/peerj.20862 (PMC13003947; doi:10.7717/peerj.20862)
Supplement: Supplemental Information 3 — Included independent variables: SST –sea surface temperature; SST1 –sea surface temperature lagged one month; SST2 –sea surface temperature lagged two months; SST3 –sea surface temperature lagged three months; SST4 –sea surface temperature lagged four months; Air temperature; Precipitation; Chlorophyll a; Wind direction and wind speed. [file peerj-14-20862-s003.docx]

**Table S2: Adjustment measure (QIC) of several Generalized Estimation Equation (GEE) models with Catostylus tagi as dependent variable.**

Included independent variables: SST – sea surface temperature; SST1 – sea surface temperature lagged one month; SST2 – sea surface temperature lagged two months; SST3 – sea surface temperature lagged three months; SST4 – sea surface temperature lagged four months; Air temperature; Precipitation; Chlorophyll a; Wind direction and wind speed.

| Included variables | QIC |
| --- | --- |
| SST | 4660.744 |
| SST1 | 4130,.66 |
| SST2 | 3624.640 |
| SST3 | 3636.024 |
| SST4 | 4161.249 |
| Air temperature | 4379.915 |
| Precipitation | 4543.324 |
| Clorophyll a | 4639.072 |
| Wind direction | 3711.481 |
| Wind speed | 3197.873 |
| SST2 + Wind speed | 2621.800 |
| SST2 + SST3 + Wind speed | 2587.246 |
| SST2 + SST3 + Wind speed + Wind direction | 2612.495 |
