# Supplementary material for: Environmental drivers of Catostylus tagi polyp survival and reproduction: unlocking the role of temperature and salinity, supported with citizen science data
Source: PeerJ. 2026 Mar 17;14:e20862. doi: 10.7717/peerj.20862 (PMC13003947; doi:10.7717/peerj.20862)
Supplement: Supplemental Information 4 [file peerj-14-20862-s004.docx]

**Table S3: Results from the generalized estimating equation (GEE) model selected.**

| Variable | B | Wald | d.f. | p-value | EXP(B) |
| --- | --- | --- | --- | --- | --- |
| Wind speed | -0.636 | 32.572 | 1 | <0.001 | 0.529 |
| SST2 | 0.613 | 63.549 | 1 | <0.001 | 1.847 |
| SST3 | 0.286 | 7.796 | 1 | 0,005 | 1.331 |
| Intercept | -10.499 | 200.026 | 1 | <0.001 | <0.001 |
